# Artificial intelligence with deep learning in nuclear medicine and radiology

**DOI:** 10.1186/s40658-021-00426-y

**Published:** 2021-12-11

**Authors:** Milan Decuyper, Jens Maebe, Roel Van Holen, Stefaan Vandenberghe

**Affiliations:** Department of Electronics and Information Systems, Ghent University, Ghent, Belgium

**Keywords:** Artificial intelligence, Deep learning, Nuclear medicine, Medical imaging, Radiology

## Abstract

The use of deep learning in medical imaging has increased rapidly over the past few years, finding applications throughout the entire radiology pipeline, from improved scanner performance to automatic disease detection and diagnosis. These advancements have resulted in a wide variety of deep learning approaches being developed, solving unique challenges for various imaging modalities. This paper provides a review on these developments from a technical point of view, categorizing the different methodologies and summarizing their implementation. We provide an introduction to the design of neural networks and their training procedure, after which we take an extended look at their uses in medical imaging. We cover the different sections of the radiology pipeline, highlighting some influential works and discussing the merits and limitations of deep learning approaches compared to other traditional methods. As such, this review is intended to provide a broad yet concise overview for the interested reader, facilitating adoption and interdisciplinary research of deep learning in the field of medical imaging.

## Background

Artificial intelligence (AI) has seen rapid progress over the last few decades, made possible due to the ever increasing amount of computational power, novel algorithms and available data. This growing amount of data is witnessed across all industries, including health care. All kinds of patient data are recorded and stored into electronic health records such as laboratory results, reports, DNA analysis, and activity and health data from wearables. A major volume of healthcare data comes from medical imaging. Due to advances in medical image acquisition, novel imaging procedures are introduced and the amount of diagnostic imaging procedures is growing fast [1]. From 2D X-rays in the early days, medical imaging evolved to multimodal, dynamic and 3D computed tomography (CT), magnetic resonance imaging (MRI), single-photon emission computed tomography (SPECT) and positron emission tomography (PET) examinations. This rising amount and complexity of imaging data increases the workload of radiologists. The Royal College of Radiologists, for example, has warned of shortages in the radiology workforce growing every year [2]. Radiologists struggle to meet the rising demand for imaging examinations resulting in delayed diagnoses and potentially affecting the accuracy of clinical decisions.

**Fig. 1:**
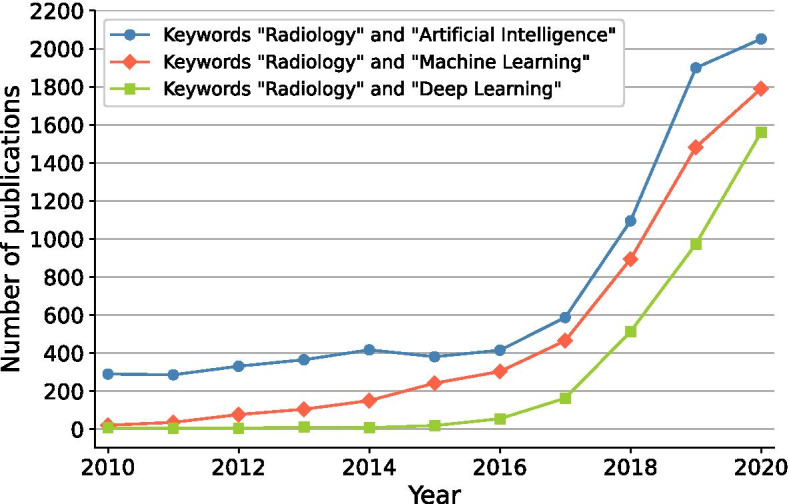
Growth of AI in radiology reflected by the number of publications on PubMed when searching on the terms “radiology” with “artificial intelligence,” “machine learning” or “deep learning”

At the same time, the increasing amount of healthcare data contains a wealth of information that presents opportunities for personalized and precision medicine. As the huge amount of data is overwhelming for physicians, we need sophisticated AI algorithms to exploit all this information. A key requirement to develop these AI algorithms is sufficient training data. Hence, the rising amount of healthcare data not only exerts great pressure on the medical industry, but simultaneously provides the opportunity to revolutionize health care. In the case of medical imaging, artificial intelligence can be employed to improve the entire imaging pipeline. This is also reflected in the amount of publications about AI in radiology on PubMed as shown in Fig. 1. AI can be applied during image acquisition and reconstruction to advance image quality, acquisition speed and reduce costs. Moreover, it can be used for image denoising, registration and translation between different modalities. Finally, a lot of AI applications are developed for medical image analysis including abnormality detection, segmentation and computer-aided diagnosis.

Medical image analysis is, however, complex. The imaging data are often 3D which adds an additional dimension of complexity. They can have large variations in resolution, contain noise and artifacts, and lack contrast which influences the performance of AI algorithms. Many applications also require information from multiple images combining different contrasts, functional and anatomical information or temporal behavior. All these elements pose specific challenges to the design of medical image analysis tools. Moreover, detection, segmentation and interpretation of anatomical structures, both normal and pathological, are inherently very complex. They have varying shapes, intensities and show large inter- and intra-subject variability. AI systems need to be robust to perform well under this wide variety of conditions.

In this article, we provide a review of different deep learning methodologies used in nuclear medicine and radiology. Section "Deep learning" provides a technical background on deep learning and the general training procedure, with special attention given to a specific type of network used in image related tasks: the convolutional neural network. In section "Medical image acquisition and reconstruction," we take a look at how deep learning can be utilized throughout the image acquisition pipeline, from improving detector capabilities to dedicated post-processing procedures. Section "Medical image analysis" provides an overview of how deep learning can help with image analysis, including image segmentation and disease detection/diagnosis. Finally, we finish with some concluding remarks in section "Conclusions."

**Fig. 2:**
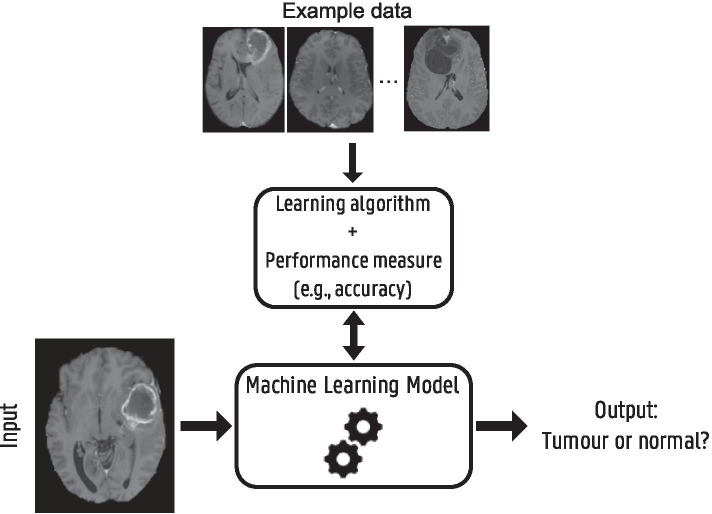
Schematic overview of different machine learning components and their interaction for a brain tumor detection example. A model, defined up to some parameters, receives a brain MRI as input and needs to provide as output whether the brain scan shows a tumor or not. Based on example data, i.e., labeled brain MRI, a learning algorithm optimizes the model parameters to improve a certain performance measure. When training is finished and the model achieves sufficient performance, it can be used to detect tumors in new MRI scans

**Fig. 3:**
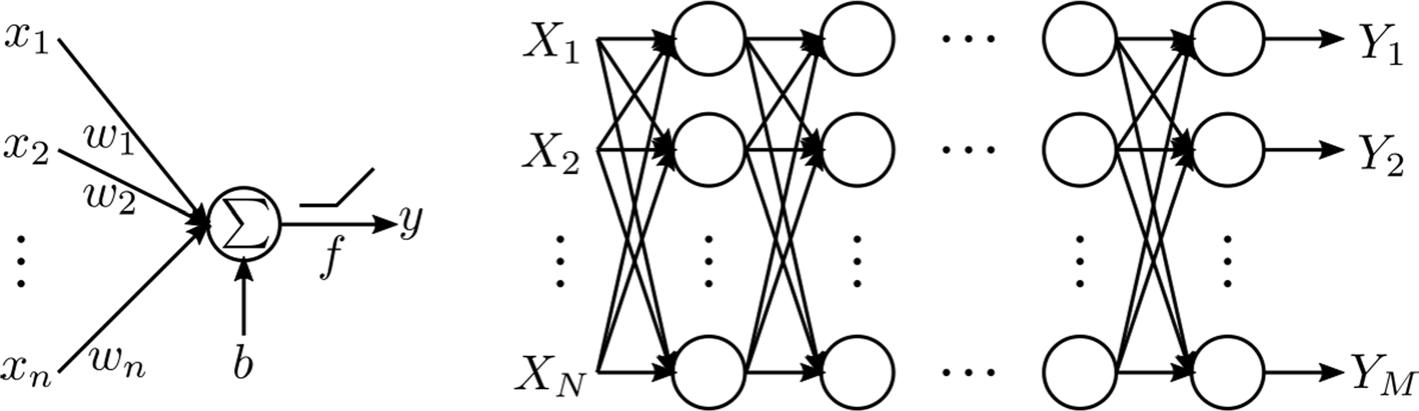
Schematic of a fully connected neural network. ${left}:$ An artificial neuron or perceptron, where the output *y* is calculated as a sum of weighted inputs ${x} =[x_1,x_2,...,x_n]$ (with weights ${w} =[w_1,w_2,...,w_n]$) and a bias *b*, optionally passed through an activation function *f*. ${right}:$ The fully connected neural network is created by connecting these neurons into many layers, where the outputs of one layer serve as the inputs to the following layer. The network depicted here consists of N inputs and M outputs

**Fig. 4:**
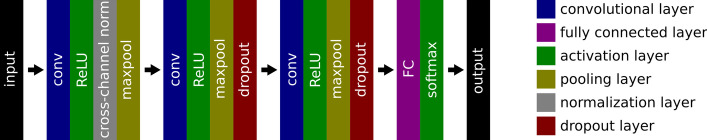
Network architecture used in [14] for brain tumor classification in MRI

**Fig. 5:**
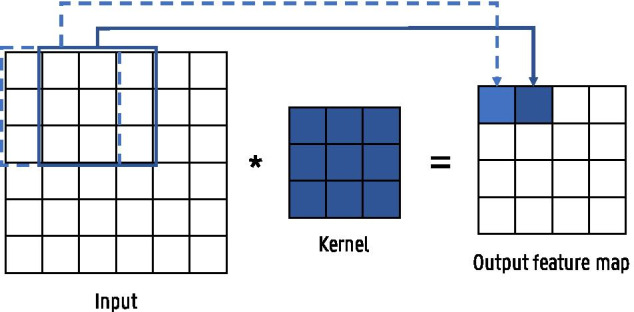
Illustration of a convolution operation between a 2D input and a kernel with size = 3 and stride = 1

**Fig. 6:**
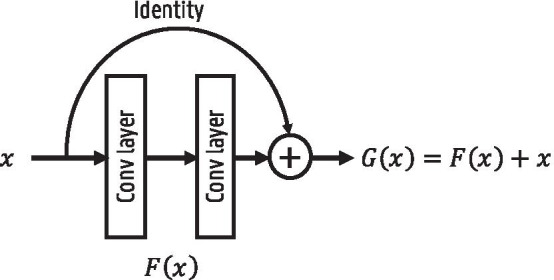
Illustration of a residual block

**Fig. 7:**
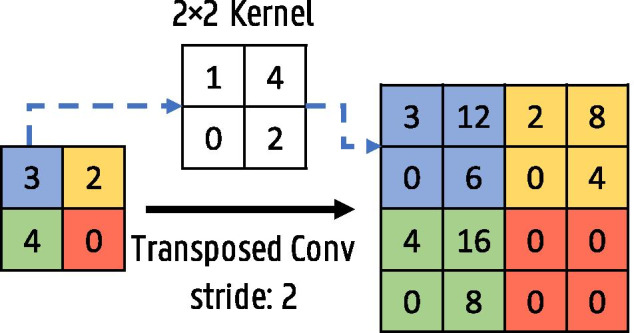
Transposed convolution operation with a $2\times 2$ kernel and stride 2

**Fig. 8:**
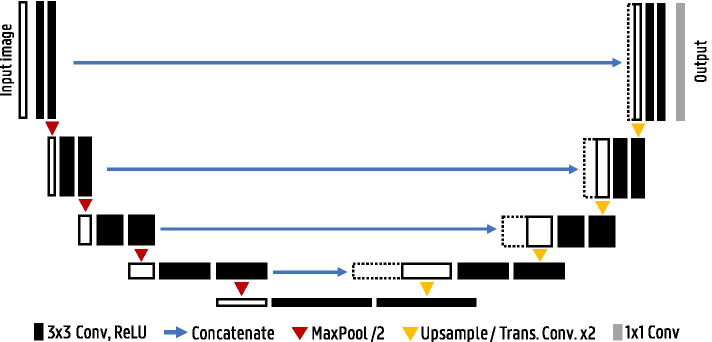
U-Net architecture

**Fig. 9:**
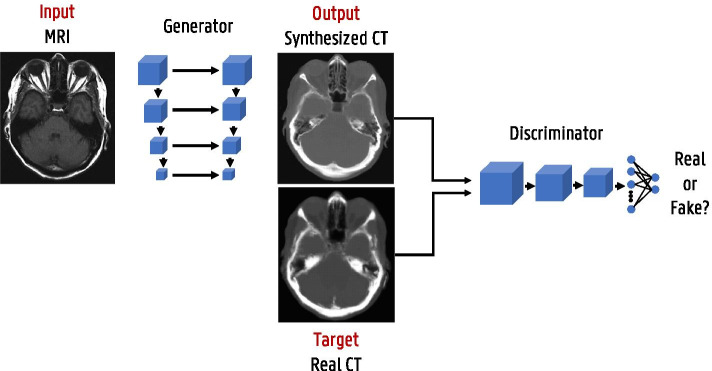
Generative adversarial network (GAN) framework illustrated with a pseudo-CT from MRI generation example

**Fig. 10:**
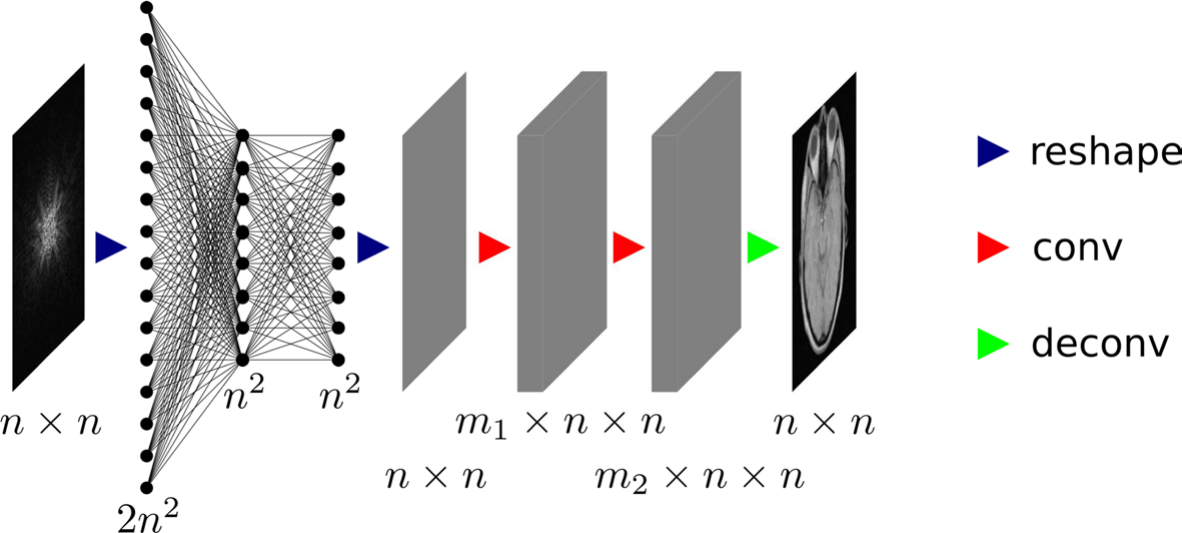
Architecture of AUTOMAP. Note that the original $n \times n$ k-space data are complex-valued, so that it is reshaped to a vector of size $2n^2$. The convolutional layers use $m_1$ and $m_2$ feature maps, respectively

**Fig. 11:**
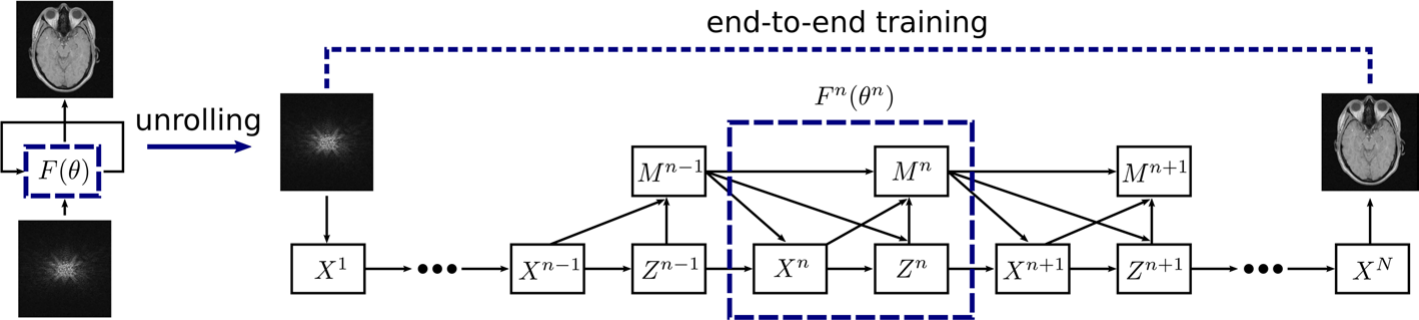
Data flow graph for ADMM-CSNet, an unrolled version of the ADMM algorithm used in compressed sensing MRI. The iterative updates $F(\theta )$ are unrolled in a neural network with fixed number of iterations. Each update block $F^n$ can have its own unique parameters $\theta ^n$, which are learned in an end-to-end fashion

**Fig. 12:**
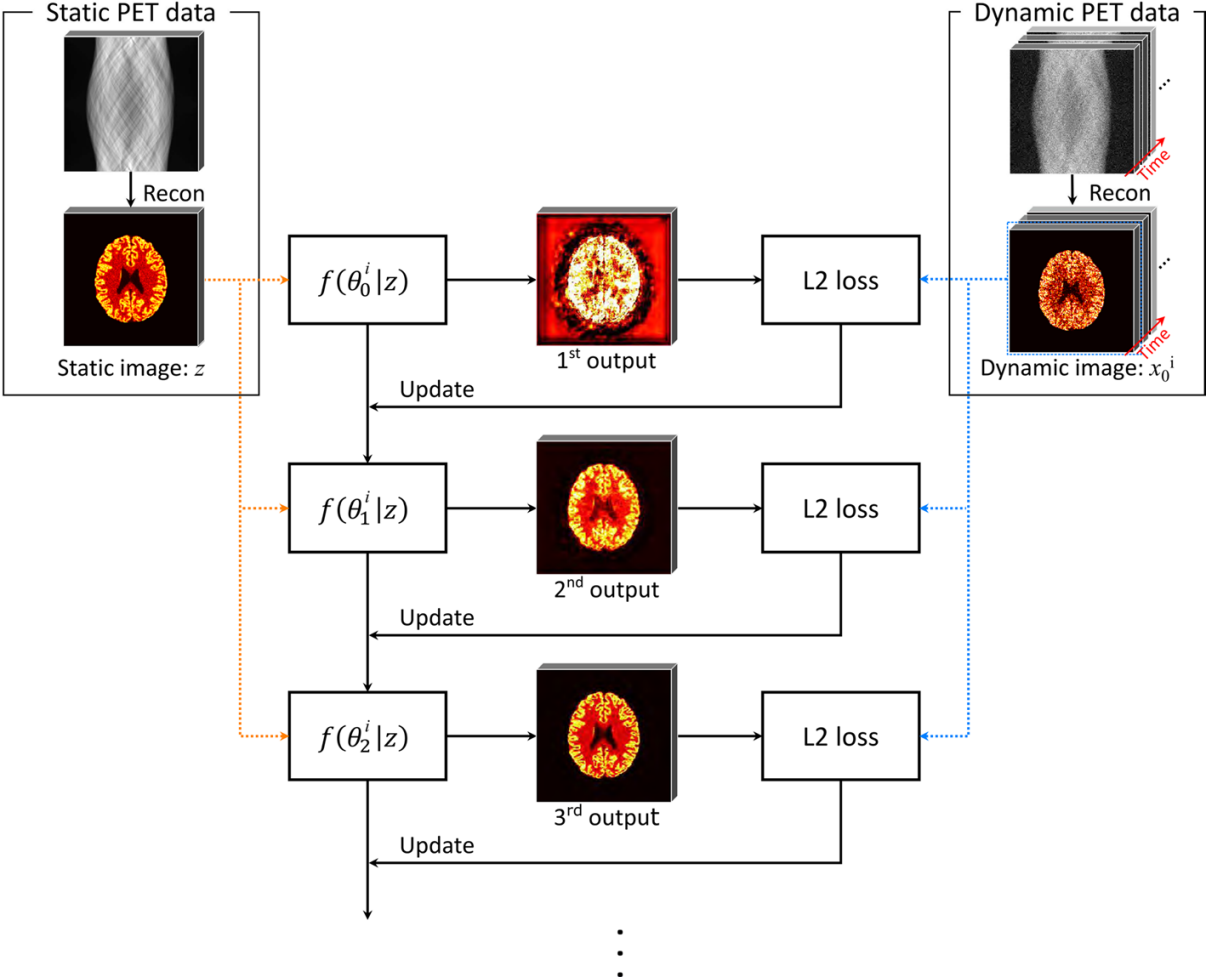
Illustration of the deep image prior training procedure for dynamic PET denoising. A static image is used as the input ${z}$ to a network *f*, initialized with random weights $\theta$. The network parameters are then iteratively optimized to produce the dynamic image *x*. After a certain number of iterations, denoised versions of the dynamic PET images are obtained as output. Image from [83]

**Fig. 13:**
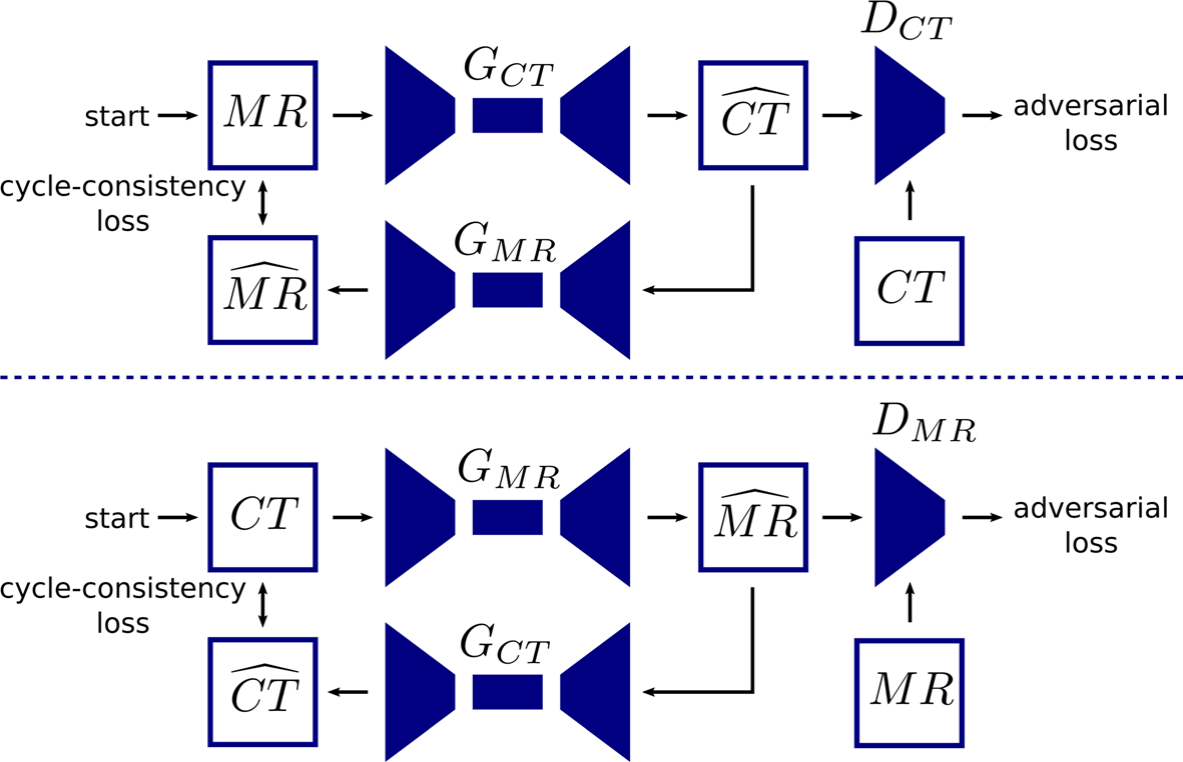
Schematic overview of a CycleGAN used for synthetic CT generation from MR

**Fig. 14:**
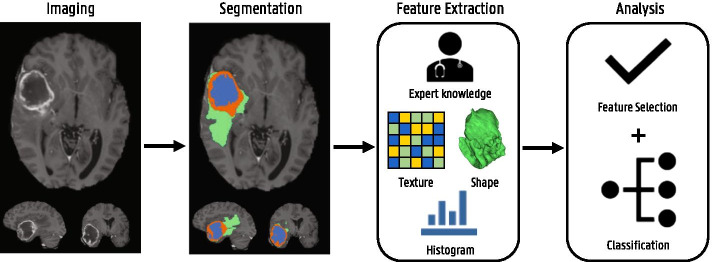
Illustration of the radiomics workflow

**Fig. 15:**
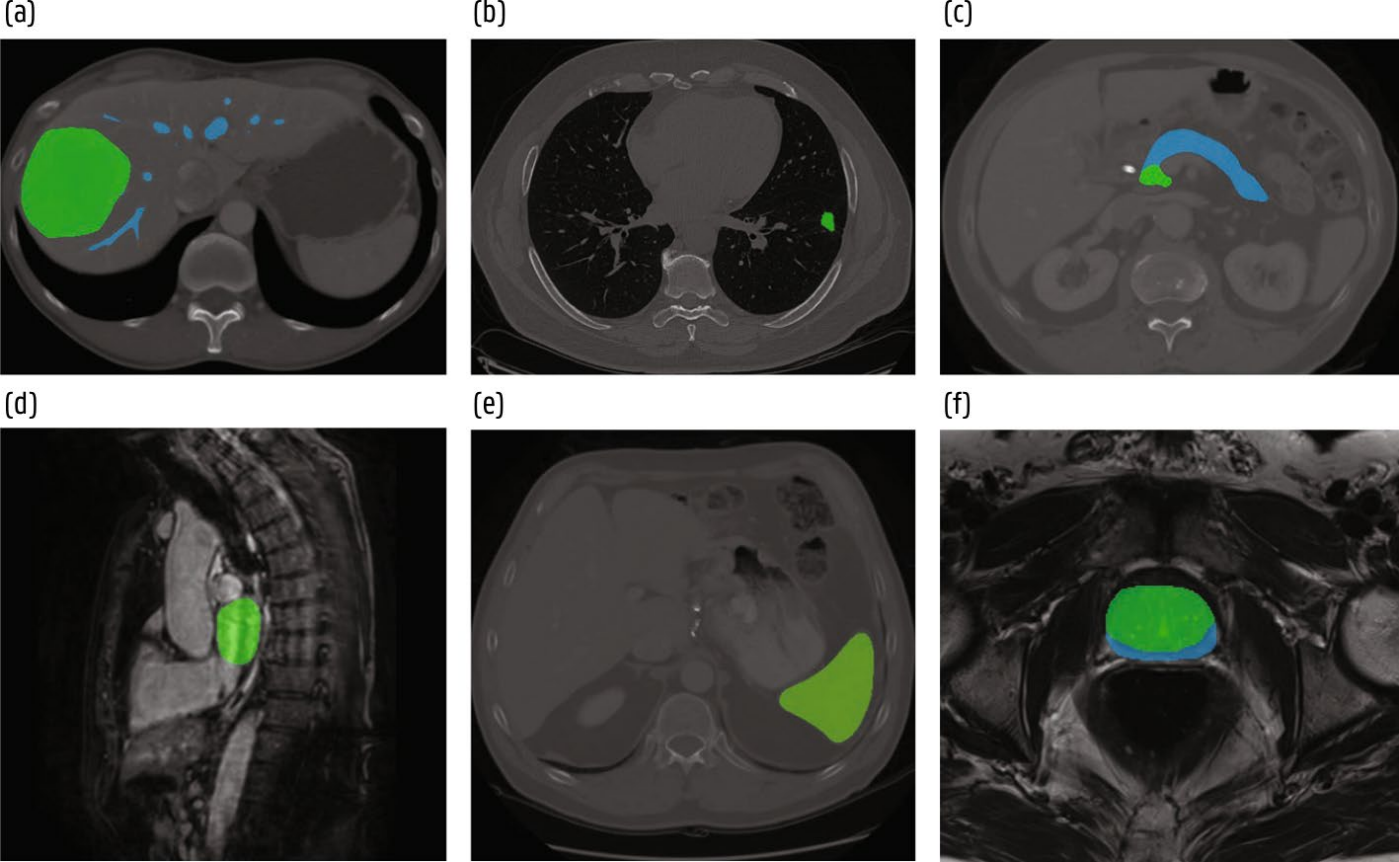
Segmentation examples from the Medical Segmentation Decathlon [134]. **a** Hepatic vessel (blue) and tumor (green) in CT. **b** Lung tumor (green) in CT. **c** Pancreas (blue) and tumor (green) in CT. **d** Left ventrical (green) in MRI. **e** Spleen (green) in CT. **f** Prostate peripheral (blue) and transitional (green) zones in MRI

**Fig. 16:**
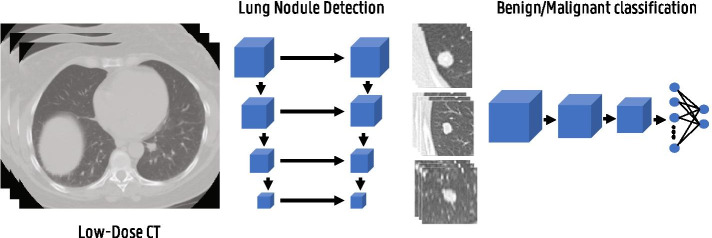
Illustration of a typical lung cancer screening pipeline consisting of a lung nodule detection and a malignancy classification stage

**Fig. 17:**
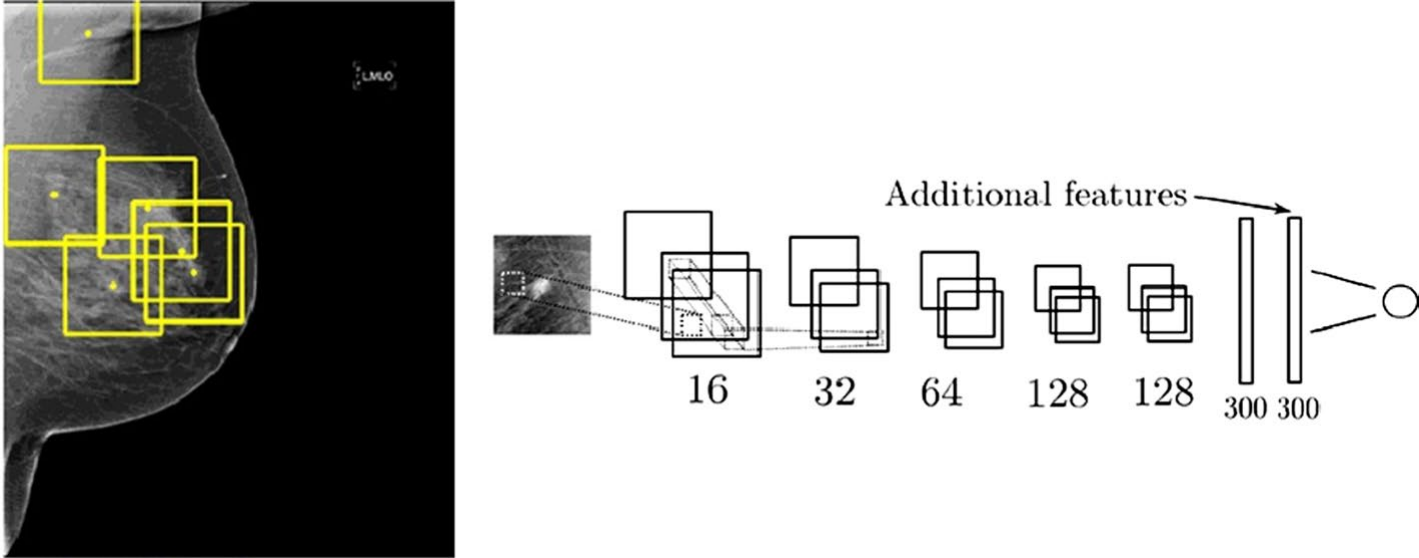
Breast cancer mammography screening using a convolutional neural network. Image adapted from [161] with permission from Elsevier

**Fig. 18:**
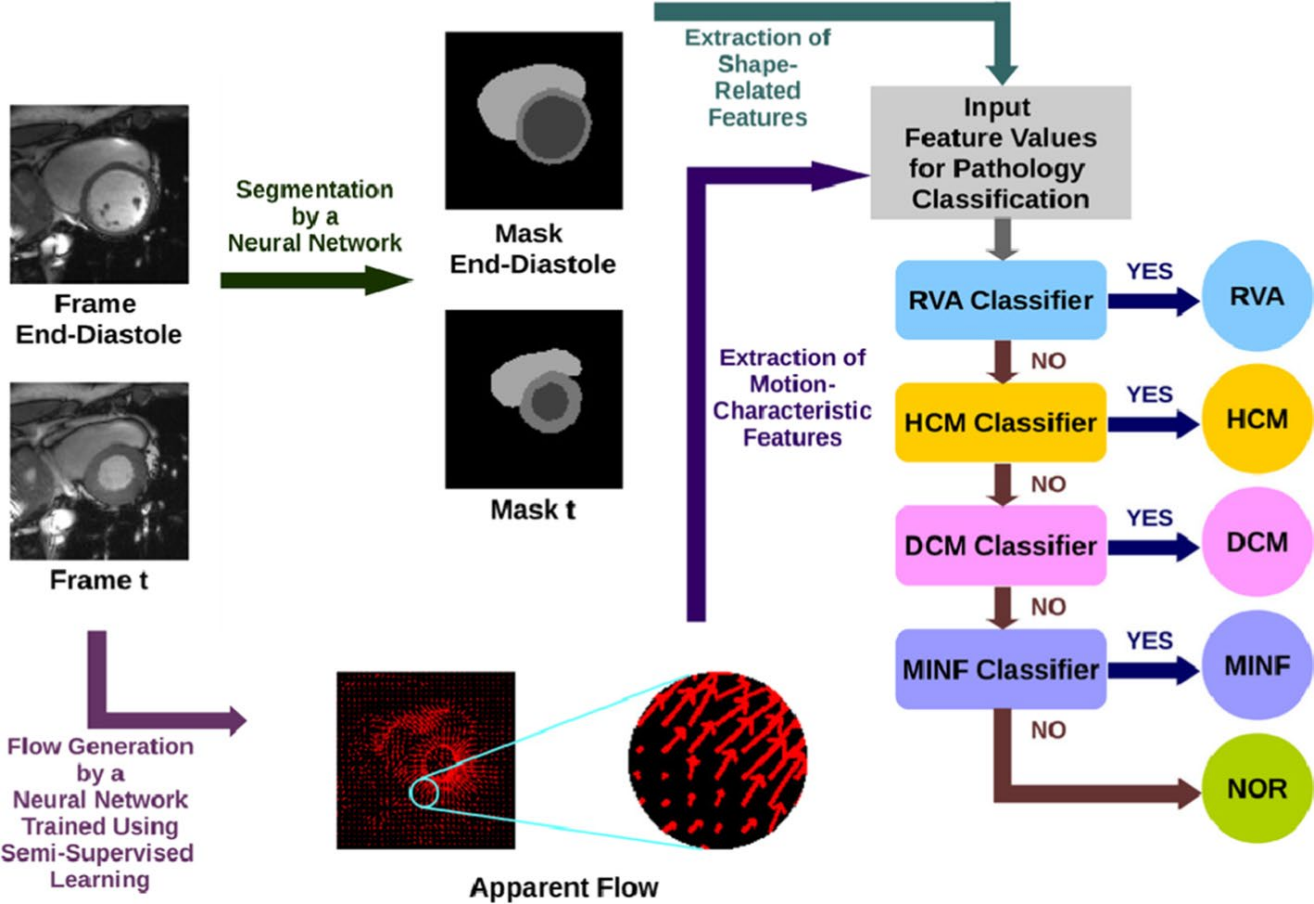
Cardiac pathology classification on cine MRI with motion characterization. Image from [173] with permission from Elsevier

**Fig. 19:**
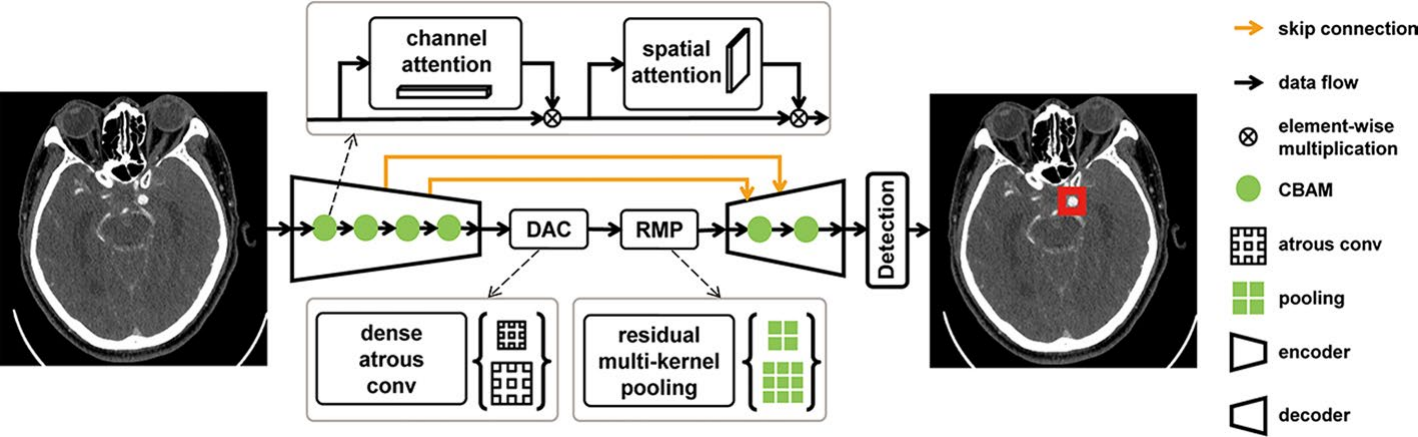
Aneurysm detection network proposed in [200]. Reproduced with permission from The Radiological Society of North America. Image from Yang J, Xie M, Hu C, et al. Deep Learning for Detecting Cerebral Aneurysms with CT Angiography. *Radiology* 2021;298:155–163

## Deep learning

This section serves as a short introduction to the domain of deep learning, covering some background and terminology which will be relevant for the rest of the article. For a more in-depth review, we refer the reader to [3]. Deep learning is a subtype of machine learning, a collective term for algorithms that are trained using example data or past experiences to perform a specific task without the need to be explicitly programmed. Figure 2 shows a schematic overview of different machine learning components illustrated with a brain tumor detection example. Based on the type of example data and available information, we can define different types of machine and deep learning.

*Supervised learning* In supervised learning, the most common type of machine learning, example data consist of known input–output pairs. Labeled data are available, and the model is trained such that its output is as close as possible to the desired label for every input. After training, the model can be applied to new unlabeled input data.

*Unsupervised learning* The second type of machine learning is unsupervised learning, where no output labels are available. The aim is to find hidden structure in the input data, for example, clustering algorithms that divide the data into groups of similar inputs.

*Reinforcement learning* The final type of learning is often used in game playing or robot control and is called reinforcement learning [4]. Here, an artificial agent learns a policy on which actions to take in an environment in order to reach a certain goal or maximize a cumulative reward. There is not one sequence of best actions, but an action is good if it is part of a good policy that in the end leads to a maximal reward. The agent explores the environment and possible actions using trial and error. Based on past good action sequences, the agent can learn a good policy.

### Artificial neural networks

Deep learning is inspired by the biological functioning of the brain, in which networks of simple interconnected processing units called neurons are used to model complex functions [5, 6]. These artificial neurons or perceptrons take an input $\mathbf{x }=[x_1,x_2,...,x_N]$, multiply it with weights $\mathbf{w }=[w_1,w_2,...,w_n]$ and sum these weighted inputs with a possible bias *b*. This result is then passed through an activation function *f* to produce an output *y* [7]:

1 $$\begin{aligned} y = f\left( \sum _{i=1}^{N}{w_ix_i}+b\right) \end{aligned}$$

Multiple neurons can be connected in layers to form a neural network, where the outputs of one layer serve as the inputs to the following layer, producing a mapping from input to output, see Fig. 3. The role of activation functions is to introduce nonlinearities in the network, required to model nonlinear relationships between input and output. A common activation function is the rectified linear unit (ReLU) [8], which simply sets negative output values to zero. Other popular activation functions are the sigmoid, hyperbolic tangent and leaky ReLU [9].

#### Training procedure

The goal of training a neural network is to find weights $w_i$ and biases *b* for each neuron so that the network correctly transforms the input data into the desired output. This is normally done using gradient descent-based methods. The weights are first randomly initialized and then iteratively optimized in three steps: forward propagation, backward propagation and a weight update. During forward propagation, input samples, usually grouped in batches, are propagated from the input, through the hidden layers to the output layer of the network. A performance metric or loss is calculated between the output predictions and the ground truth labels. This loss is then back-propagated from the output layer to the input where, at every layer, the gradient of the loss with respect to the weights is computed using the chain rule [10]. The weights are then updated using the negative gradient with a certain step size or learning rate. Many gradient descent-based methods exist, with a popular and robust optimization algorithm being the adaptive moment estimation (Adam) algorithm [11]. It uses a combination of momentum and adaptive learning rates for individual parameters, resulting in generally faster convergence speeds than standard stochastic gradient descent.

In addition to the learnable network weights and biases, there is also a large amount of hyper-parameters which need to be set prior to network training. These include the network architecture and size, choice of activation function, choice of optimization algorithm, batch size, learning rate, etc. Many of these parameters can have a large impact on convergence speed and final network performance and need to be fine-tuned. This has also led to the development of algorithms to facilitate and automate the optimization of hyper-parameters [12].

#### Convolutional networks

The network shown in Fig. 3 is a fully connected network, where all neurons of one layer are connected to all neurons of the following layer. When dealing with structured input data, however, such as 2D or 3D images in nuclear medicine, convolutional neural networks (CNNs) [13] are commonly used instead. We will further discuss these using a specific example from supervised learning: a CNN for brain tumor classification in MRI [14]. In this study, T1-weighted contrast enhanced MR image slices showing a brain tumor are used as the input for a CNN, predicting a tumor classification (tumor type or grade). The network architecture is depicted in Fig. 4, where we can distinguish several layers, each serving a specific role.

##### Convolutional layers

We notice three convolutional layers throughout the network. These layers consist of several kernels, containing the trainable weights or parameters of the layer, that are convolved with the input. They have the same number of dimensions as the input with an equal depth, but are usually much smaller in the other dimensions. The kernel size determines their receptive field. Figure 5 illustrates a 2D convolutional operation with a depth of 1. The kernel size is set to a width and height equal to 3 resulting in a receptive field of $3\times 3$. The kernel slides over the entire input with a predefined step size or stride, and at every position, a dot product is performed between the kernel and the current input patch. This way, a feature map is created containing the output responses of the kernel at every spatial position. Every convolutional layer consists of several kernels and produces an equal amount of feature maps. The motivation behind using convolutional layers is twofold: sparse connectivity and parameter sharing.

Sparse connectivity means that, in contrast to fully connected layers, the output neurons are not connected to all input units. Input images can contain millions of pixels, so instead of connecting a neuron with every input pixel, relevant features such as edges can be detected using kernels that are much smaller than the input. Although the receptive field of each kernel is small, deeper layers that interact with multiple outputs of earlier layers have an increasingly large receptive field with respect to the input. This allows the network to model complex interactions between simple building blocks across the input.

Parameter sharing denotes that the same kernel is used multiple times across the entire input, while in a fully connected network each weight is only used once. Consequently, a feature only needs to be learned once instead of multiple times for every location. Parameter sharing also causes a convolutional layer to be translational equivariant. This means that, if the input is translated, the output translates in the same way. This is especially useful when features, which detect edges for example, are relevant across the entire input. Moreover, because of parameter sharing, the input size does not have to be fixed which allows to process inputs with varying sizes.

Sparse connectivity and parameter sharing result in a large reduction in number of parameters which improves statistical efficiency and reduces memory requirements and amount of computations [3].

##### Fully connected layers

In our example, we see a single fully connected layer after the convolutional blocks. One or more of these are normally applied to the end of a CNN in specific tasks such as classification or regression problems. They use the features extracted by the convolutional layers to determine the final output. Hence, the convolutional layers are generally seen as the feature extractors of the CNN and the fully connected layers as the classifier or regressor.

*Activation layers*  There are two different activations used in Fig. 4. The first is a ReLU activation following the convolutional layers, whose role is to introduce nonlinearities in the network. The final activation, however, serves a different purpose. It is a softmax layer that produces the final output, ensuring that the values remain contained within the desired range. Since we are dealing with a multi-classification problem, the softmax layer produces output values between 0 and 1 (one output value for each class), representing the probability of belonging to that particular class. It also ensures that the sum of output values equals 1. Different output activation functions may be used depending on the problem, e.g., a sigmoid activation in the case of binary classification, no activation in the case of a regression, or a clipping function in the case of image outputs where all pixels should be between 0 and 1.

*Pooling layers* Pooling or subsampling layers reduce the size of the input by calculating summary statistics over a predefined neighborhood. As the number of parameters in the next layers depends on the input size, pooling allows to improve the computational efficiency and reduce memory requirements. Our specific example used max-pooling layers, but different statistics such as average pooling are also possible. The neighborhood size is usually set to $2\times 2$, effectively reducing the input size by half. Alternatively, the pooling operation can also be performed using convolutional layers with a stride larger than one.

*Normalization layers*  The weights of every layer are updated based on the assumption that the other layers do not change. Changes to the early layers will, however, affect the deeper layers. To minimize this effect, a normalization can be introduced, ensuring that the input of each layer is re-normalized to zero mean and unit variance. Our example uses a cross-channel or local response normalization layer (normalization across the features in a single training example), but another popular choice is batch normalization (normalization across the different samples of a feature in a single training batch) [15]. These normalization layers can be used after any convolutional or fully connected layer, either right before or after the activation layer.

*Dropout layers* A dropout layer randomly sets input elements to zero during training, but does nothing during testing. This is done as a form of regularization and will be discussed in more detail in the following section.

### Generalization and regularization

The versatility of deep learning lies behind the universal approximation theorem, stating that feedforward networks with at least one hidden layer, using a nonlinear activation and a linear output layer, can approximate any continuous function [16, 17]. That is, these deep learning models should be able to fit any sufficiently well-behaved training data to arbitrary precision by expanding the hidden layer size, thereby allowing the network to model increasingly complex functions. One of the main challenges in machine learning is, however, to train a model that not only performs well on training data, but also on new, unseen data. This is called generalization. To assess the generalization performance of a model, the available data are typically split into a training, validation and test set. The training set is used to optimize the model weights, whereas the validation set is used to evaluate the generalization performance of the model during training. Hence, no weights of the model are optimized using validation data but the model hyper-parameters are tuned to minimize the validation error. After training the model is finally evaluated on the test set to assess the predictive power on unseen samples.

One way to achieve generalization in deep learning is to reduce the model capacity by, e.g., limiting the number of neurons or layers in the network. When a model is too complex, it can have a tendency to overfit on the training data, resulting in poor test performance. Reducing the model capacity too much is, however, also detrimental, as the model will underfit and result in poor performance on both training and test sets. In practice, it is often beneficial to use deeper models with less neurons per layer to achieve better generalization without underfitting.

Instead of changing the variety of functions that the model can represent, we can also incorporate a preference toward certain functions to limit the amount of overfitting. This is called regularization or *“Regularization is any modification we make to a learning algorithm that is intended to reduce its generalization error but not its training error”* [3]. We will now discuss a number of regularization techniques applicable to deep neural networks.

*Data augmentation* The best strategy to reduce overfitting is to train the model on more data. Of course, in practice the amount of available training data is limited and it is not always possible to collect new additional data, especially in a medical context where data annotation is labor-intensive and requires expert knowledge. Data augmentation allows to artificially create new data samples based on the existing training set [18]. Most data augmentation techniques are based on transformations or alterations that the model should be invariant to. For example, the aforementioned brain tumor classification example used up–down flipping, left–right mirroring, addition of salt noise and 45$^{\circ }$ rotations for data augmentation.

*Early stopping* When training neural networks, we typically observe a behavior where the training error steadily keeps decreasing while the validation error starts to increase again after some time. Therefore, instead of training a neural network for a fixed number of iterations, it can be beneficial to monitor the validation error during training and terminate the training process when no further improvement of the validation loss is observed for a predefined number of iterations. The optimal network state is then chosen at the point in time where validation error was lowest. This strategy is known as early stopping.

*Dropout* Another regularization technique, effective in a lot of application domains, is dropout [19, 20]. Here neurons of the network are randomly dropped during training with a certain probability. Hence, for every sample in the mini-batch, different units are set to zero and a different subnetwork is created. Therefore, dropout can be thought of as a way to create and train an ensemble of many subnetworks and thereby improve the generalization performance. Another view on why dropout has a regularizing effect is that it prevents coadaptation of different neurons. By removing different neurons at every iteration, neurons that are included should perform well regardless of which other neurons are included in the network. Hence, it forces the neurons to be relevant in many contexts. Our example in Fig. 4 used two dropout layers, with dropout probabilities of 10% and 20%.

*Cost function penalty* Different cost functions are used for different prediction tasks; for example, the cross-entropy loss is commonly used for multi-classification problems. Our brain tumor classification example, however, added an additional penalty term to the cost function, namely the $l_2$ norm of the layer weights $w_i$. This has a regularizing effect, as the training procedure now results in overall lower weight values, leading to a simpler, and therefore more generalizable, model. The $l_1$ norm is another commonly used cost function penalty, promoting sparser solutions.

*Transfer learning* Transfer learning refers to techniques where knowledge learned from one task is transferred to another task instead of training a network from scratch [21]. It is expected that features learned to identify, for example, cats and dogs in images can be applied to other image recognition tasks as well. This is especially useful in case only a small amount of data is available for the new target task. Through the use of a good starting point, i.e., a network pretrained on a different related task for which a lot of data are available, high performances can be achieved with only a limited amount of data.

### Key architectures for medical imaging

To conclude this introductory section to deep learning, we will discuss a selection of key CNN architectures prevalent in medical imaging applications, which will often be referred to throughout the rest of this paper.

#### ResNet

The ResNet architecture was proposed for image classification tasks [22]. Earlier works indicate that increasing network depth strongly improves the image recognition capacity. It was found, however, that when further adding additional convolutional layers the training accuracy saturated and even started to degrade. As this behavior was observed on the training accuracy, it was not caused by overfitting. This shows that current optimizers find it hard to train increasingly deep networks. A deeper model that performs equally well as its shallower counterpart should exist, as it can be constructed by adding layers performing an identity mapping to the shallow network. Based on this idea, skip connections or residual blocks were introduced in [22]. The residual block is depicted in Fig. 6. Instead of directly learning the underlying mapping *G*(*x*), the layers learn the residual $F(x) = G(x) - x$ due to the skip connection. Their results show that it is easier to optimize the residual function than the original mapping. Hence, skip connections allow for better optimization of deeper networks.

#### U-Net

In 2015, U-Net was proposed as a biomedical image segmentation architecture [23]. The authors employed the architecture in several segmentation challenges such as segmenting neuronal structures in electron microscopy stacks or cell segmentation in light microscopy images and won with a large margin.

The typical use of CNNs was to classify an entire image into a single class label. In many computer vision tasks, however, localization is required where every pixel is labeled with the class of the object it belongs to. These so-called semantic segmentation tasks were usually tackled using standard classification CNN architectures. Each pixel is separately classified by providing a local region (also called patch) around the pixel to the classification network. Using a sliding-window approach all pixels of an image are classified. This approach has the advantage that additional training data can be generated as a lot of patches can be extracted from one image. This is especially useful in biomedical tasks where the amount of training data is often limited. There are, however, two drawbacks to this strategy. First of all, segmentation of an image is inefficient as many overlapping patches need to be propagated through the network. Secondly, finding the optimal patch size is difficult due to the trade-off between larger patches containing more context and smaller patches for better localization.

To combine both context and good localization accuracy, the fully convolutional network was introduced [24]. The idea is to add upsampling layers after the usual contracting classification network to increase the resolution of the output back to the input image resolution. No fully connected layers are used to preserve spatial information. To increase the output resolution, simple bilinear upsampling can be employed. Another approach is to use transposed convolutions, also called up- or deconvolutions, where the upsampling parameters are learned. The output size of the transposed convolution layer depends on the chosen kernel size and stride. A transposed convolution operation with a stride of two and kernel size $2\times 2$ is illustrated in Fig. 7.

In the U-Net architecture this upsampling path is further extended with convolutional layers, allowing to propagate context information to the higher-resolution layers [23]. This results in a more or less symmetric U-shaped architecture with a contracting and expansive path (see Fig. 8). This type of architecture is also called an encoder–decoder network. To improve localization, skip connections are added between the high-resolution features of the encoder path and the upsampled feature maps in the decoder path. U-Nets efficiently use semantic and spatial information for accurate segmentation and are still the state of the art for many segmentation tasks.

#### GAN

State-of-the-art deep learning solutions for image-to-image translation tasks mostly use an image generation network (such as the aforementioned U-net) combined with a discriminator network to form generative adversarial networks (GAN) [25]. Generative adversarial training is a framework where two networks, a generator and a discriminator, are simultaneously trained to compete against each other [26, 27]. This is illustrated with a pseudo-CT from MRI generation example in Fig. 9. The generator focuses on image synthesis and tries to fool the discriminator which is trained to identify real versus synthesized images. While training, the gradients are back-propagated from the discriminator to the generator, so that the parameters of the generator are adapted to produce realistic images according to the discriminator. Next to this adversarial loss other loss functions such as $l_1$ loss are incorporated as well to retain image details. GANs and variants thereof, e.g., cycleGAN [28], are widely used in image reconstruction and enhancement.

## Medical image acquisition and reconstruction

This section delves into the use of deep learning during the imaging chain, a broad topic covering various aspects such as detector performance, image reconstruction and advanced post-processing. While the focus remains on deep learning-based algorithms, we will also discuss conventional algorithms where relevant, in order to highlight some of the key differences in terms of implementation and capabilities.

### PET and SPECT detectors

At the heart of the acquisition process lie the detectors, collecting the data which is later converted into human-interpretable images. Improvements made early on in the acquisition chain have a propagating effect throughout the entire imaging procedure, from reconstruction to analysis, ultimately enhancing diagnostic performance. Unfortunately, one is often constrained by inherent physical process limitations or current material technologies. Furthermore, there is a trade-off between scanner cost and performance that should be taken into account for practical purposes. We take a closer look at PET and SPECT gamma detectors to see how these can benefit from deep learning to more effectively use data already available to us.

Most PET or SPECT detectors make use of scintillation crystals, which absorb the gamma photon by the photoelectric effect and re-emit its energy as visible light. These optical photons are then detected by photomultiplier tubes (PMTs) or silicon photomultipliers (SiPMs) coupled to the crystal, converting the optical signals into electrical ones. Current clinical scanners make use of pixelated detectors consisting of a crystal block subdivided into an array of smaller pixels, each a few millimeters wide, with limited optical photon transfer in-between. A light guide between crystal and photomultiplier surface may be used to spread out the scintillation light, so that one-to-one coupling between pixel and SiPM is not required. Preclinical systems have also seen the development of monolithic detectors, in which a single continuous crystal a few tens of millimeters wide is coupled to an array of multiple SiPMs. Since gamma generation in PET happens through positron annihilation, the produced gamma photons always have an energy of 511 keV. SPECT, however, makes use of gamma-emitting radionuclides, resulting in isotope-dependent gamma energies, e.g., 140 keV for the commonly used isotope $^{\mathrm{99m}}\hbox {Tc}$. The crystal material and thickness can therefore vary greatly, as the detection efficiency should be optimized for the respective energy.

Digital detectors, which directly convert the gamma energy into electrical signals, have also been developed for use in SPECT but are not under consideration here. In fact, the majority of research on deep learning for scintillation detectors has been focused on PET specifically, although many results and conclusions are also applicable to SPECT.

#### Positioning

*Pixelated detectors* In pixelated detectors, the interaction pixel is easily determined by centroid weighing methods such as Anger logic. More advanced techniques such as dictionary-based algorithms or deep learning offer little advantage as the 2D resolution ultimately remains constrained by the pixel size. Improvements can, however, be made when it comes to obtaining depth-of-interaction (DOI) information normally not available in these detectors. This is of little importance for SPECT, since the collimator filters for perpendicular incidences so that the 2D position contains all necessary information. In PET, however, lack of DOI decoding leads to incorrect line-of-response (LOR) assignment for non-perpendicular coincidences, reducing image reconstruction accuracy. A possible solution is the addition of a front- or lateral-sided readout, but the added electronics increase costs and create additional dead space between detector blocks. As an alternative, a linear method was developed for continuous DOI estimation based on scintillation light sharing through a common light guide on the front surface of the crystal [29]. This was later improved upon by replacing the linear method with a neural network estimator [30]. The energies measured by the SiPM array are used as input features to predict a continuous DOI position. Both a dense neural network and a CNN were tested, showing performance similar to each other but improved by 12 - 26 % compared to the linear method. Uniformity was also significantly better throughout the crystal array.

*Monolithic detectors* Monolithic detectors on the other hand are attractive as these are not constrained by pixel size for spatial resolution and offer easier access to DOI information. Unfortunately, the aforementioned Anger logic no longer provides optimal spatial resolution. It particularly fails at the edges of the crystal due to nonlinear light distributions and leads to incorrectly predicted impinging locations for non-perpendicular incidences. Early works have shown that neural networks could offer superior spatial resolution with good uniformity and, by providing training data at different incidence angles, could predict the impinging location for non-perpendicular incidences without the need to correct for DOI [31–33]. Later works included the DOI as an additional output, allowing for 3D positioning [34–36]. Training data can be obtained by the pencil beam method or Monte Carlo simulation. The charge collected by the SiPMs, possibly obtained in a row–column summing configuration, is used as the input to a dense neural network for predicting a 2D or 3D position. Some later studies replace the dense neural network with a CNN [37]. Performance is generally improved compared to other conventional methods, with better uniformity owing to higher spatial accuracy at the crystal edges.

#### Scattering

As a gamma photon passes through a scintillation crystal, it may undergo Compton or Rayleigh scattering before photoelectric absorption in another pixel or detector block. Rayleigh scattering, an inelastic process without energy transfer, is practically undetectable as no optical photons are generated. The elastic Compton interactions on the other hand convert a part of the gamma energy, proportional to the scattering angle, to scintillation light and reduce the energy available for subsequent photoelectric absorption. Such Compton scattered events are easily observed for interactions in different crystals or pixels, but identification of the first gamma interaction remains complicated, leading to image degradation due to incorrectly assigned LORs in PET or erroneous counts in SPECT. They are therefore often discarded, resulting in a loss of sensitivity.

In [38], a neural network approach was developed for assigning the LOR in PET for triple coincidences, where one 511 keV photoelectric event *P* coincides with two more singles $S_1$ and $S_2$, whose energy sum also equals 511 keV. In ideal circumstances, it is often possible to analytically derive which single lies on the LOR by considering the relation between scattering angle and energy deposit. The limited energy resolution and positioning accuracy, however, degrade these analytical methods considerably, which the deep learning approach seeks to solve by inherently taking such limitations into account with realistic training data. The interaction coordinates of $S_1$ and $S_2$ are first redefined in a plane w.r.t. *P*, so that a dense neural network with only 6 inputs (2D coordinates of $S_1$ and $S_2$ and their measured energies) can be trained to predict which of the two scattered singles lies on the LOR. Ground truth data are provided by means of Monte Carlo simulation. This method showed a LOR recovery rate of 75%, yielding a 55% sensitivity increase when including these triple coincidences on real data from the LabPET scanner [39]. It showed acceptable resolution degradation similar to other sensitivity increasing methods with little to no contrast loss.

Monolithic detectors additionally suffer from intra-crystal scatter degradation, but as scintillation light is not confined to pixels, it becomes difficult to discern scattered from non-scattered events, let alone assign separate positions and energies to subsequent interactions within the same crystal.

#### Timing

In time-of-flight (TOF) PET, interaction timing information is used to more accurately determine the positron annihilation position along the LOR. This information can then be used during image reconstruction to improve scan quality. The ultimate objective is to reach a coincidence time resolution (CTR) of 10 ps full width at half maximum (FWHM), which would allow for millimeter level annihilation positioning so that the tracer distribution can be obtained directly without the need for tomographic reconstruction. This is still a distant objective, with current clinical TOF-PET scanners possessing a CTR of a few hundred picoseconds. Combined with detector advancements, deep learning may help to reach this goal sometime in the future.

Timing estimation is traditionally done by recording the moment the SiPM signal crosses a predefined threshold. This, however, condenses all of the potentially useful signal information into a single linear estimator. In [40], it was shown that convolutional networks could be used to predict the TOF difference directly from the detector signals themselves. The study used the outputs of two opposing detector pixels, digitized using 100 ps binning and then stacked side-by-side, as a single CNN input for predicting the TOF difference between both detectors. Only the short rising edges of the signals were used, as most of the important timing information is contained within the first few arriving scintillation photons. This method showed promising results, improving the CTR by 20% compared to leading edge detection and 23% compared to constant fraction discrimination.

### Image reconstruction

Most medical imaging modalities do not generate data directly in image space, instead requiring reconstruction algorithms to obtain sensible information. While tomography (CT, PET and SPECT) produces projections of a 3D volume and MRI generates spatial frequency data, both processes can be described in operator form as

2 $$\begin{aligned} \varvec{y} = \varvec{Ax} + \varvec{e} \end{aligned}$$

where $\varvec{y}$ is the measured data (projections in tomography, k-space data in MRI), $\varvec{A}$ is the system operator describing the physics and geometry of the imaging process, $\varvec{x}$ is the unknown image data and $\varvec{e}$ is additive noise. The inverse problem of finding $\varvec{x}$ is ill-posed and lacks an exact solution, but an analytical closed-form approximation can be found by, e.g., the filtered back-projection (FBP) algorithm for tomography or the inverse fast Fourier transform (iFFT) for MRI. Such analytical solutions are popular due to their computational simplicity, but their failure to model scanner non-idealities and noise statistics leads to inaccurate image estimations.

It is instead preferred to find a solution for equation 2 by minimizing an objective function *f* providing a measure for the reconstruction accuracy:

3 $$\begin{aligned} \hat{\varvec{x}} = \arg \min _{\varvec{x}} [f(\varvec{Ax} + \varvec{e}, \varvec{y})] \end{aligned}$$

The minimum can be found by iterative algorithms such as expectation maximization, which recursively update $\varvec{x}$ to better match the measured data $\varvec{y}$. Image quality is improved compared to analytical methods by making explicit use of the forward operator $\varvec{A}$ in each iteration, in which various physical limitations can be included. Many options exist for the cost function, and it can, for example, be chosen based on the noise statistics of the imaging modality. The $l_2$ norm is a good choice for MRI as it is dominated by white Gaussian noise, and the negative log-likelihood can be used for PET and SPECT to among other things take into account the Poisson statistics of single-photon counting and radioactive decay. Due to the ill-posedness of the problem, small perturbances in the measured data $\varvec{y}$ can lead to large changes in the image estimate $\varvec{x}$, easily resulting in an overfit on the measured data. A regularization term *R* is therefore included to penalize unlikely solutions $\varvec{x}$ based on a priori assumptions about the image properties, such as demanding smooth or low-noise solutions:

4 $$\begin{aligned} \hat{\varvec{x}} = \arg \min _{\varvec{x}} [f(\varvec{Ax} + \varvec{e}, \varvec{y}) + \lambda R(\varvec{x})] \end{aligned}$$

The optimization problem becomes a trade-off between the data consistency term *f* (how accurately the image estimate $\varvec{x}$ produces the measured data $\varvec{y}$) and the regularization term *R* (the overall noise level), the relative strength of which can be controlled by the hyper-parameter $\lambda$. There are again many options for the regularization function, a commonly used example being total variation, promoting piecewise smooth regions.

Iterative methods, while certainly an improvement over analytical ones, are not without their own drawbacks. They are computationally expensive and may still include modeling errors in the forward operator *A*, and the regularization term and its strength $\lambda$ ultimately involve user-specified assumptions about what are considered acceptable image properties. Deep learning-based approaches seek to solve these limitations by replacing the uncertain user-defined variables in traditional methods with parameters learned from data.

#### Data-driven approaches

One option for deep learning image reconstruction is to replace equation 4 with a neural network *F* that takes into account all system properties and noise statistics so that:

5 $$\begin{aligned} \hat{\varvec{x}} = F(\varvec{y}) \end{aligned}$$

The network learns to directly reconstruct the image from projection/k-space data by training on known data pairs $\varvec{x}$ and $\varvec{y}$. Convolutional encoder–decoder networks are typically used, having proved capable in various other image-to-image translation tasks [27, 41, 42]. These networks contain a contractive path, extracting (encoding) features from the input data, and an expansive path that constructs (decodes) the output from these features. They have a similar architecture to the U-Net shown in Fig. 8, but do not make use of skip connections given the large structural difference between input and output.

Once training is finished, reconstruction of new images is fast as it only requires a single forward pass through the network. These direct reconstruction methods are entirely data-driven, meaning the full inverse mapping is learned from training pairs without making any underlying assumptions about the imaging process itself. This limits modeling errors and allows the noise characteristics to be learned from data rather than being predefined by the regularization term. Learning such a complex relationship does require large amounts of training data, which can be difficult to obtain since the true image $\varvec{x}$ is generally unknown to us. Simulated data with known $\varvec{x}$ or traditionally reconstructed images with low noise levels (e.g., high-dose images) for which $\hat{\varvec{x}} \sim \varvec{x}$ may be used instead.

The prime examples of direct deep learning reconstruction are AUTOMAP (automated transform by manifold approximation) [43] for MRI and DeepPET [44] for PET. AUTOMAP proposes a generalized data-driven method for solving inverse problems. It does so by learning a mapping from sensor-domain to image-domain data, where a low-dimensional joint manifold of the data in both domains is implicitly learned during training. This low-dimensional but highly expressive representation of the data ensures robustness to noise and other input perturbations. AUTOMAP is implemented as a neural network consisting of three fully connected layers followed by a sparse convolutional autoencoder (see Fig. 10). The fully connected layers learn the between manifold projection from sensor to image domain, whereas the convolutional layers force the image to be represented sparsely in convolutional feature space. Since the mapping is learned from scratch, non-trivial acquisitions (e.g., non-Cartesian, undersampled or misaligned Fourier data) can be used directly as the input without additional preprocessing in Fourier space. The authors showed that the mapping could be learned not only from real MRI data, but also from natural or even pure noise images for which the scanner response was simulated. As the training dataset becomes more specific (from pure noise images to real MRI data), more relevant features for MRI reconstruction are extracted, leading to a lower-dimensional manifold approximation and better robustness to noise. While the methodology in the paper was shown for MRI, the authors of AUTOMAP emphasize that it is applicable to generalized reconstruction problems, and also show an evaluation on PET data. DeepPET on the other hand uses a more conventional convolutional encoder–decoder architecture. It reconstructs PET images from 2D sinograms by training on simulated PET data obtained from the humanoid XCAT (extended cardiac-torso) digital phantom [45]. The network was later also used as the generator in a Wasserstein GAN for improved reconstruction quality [46].

One common drawback of these algorithms is that fully 3D reconstruction is not possible with current GPU memory sizes, therefore remaining limited to 2D slice by slice reconstruction.

#### Model-driven approaches

Besides the large data requirements, the aforementioned approaches lack in interpretability given their black box nature and concerns remain about the generalization capability for out-of-domain cases. Such limitations have lead to an increasing interest in physics-aware deep learning, where the neural network incorporates existing domain knowledge prior to training. As a concrete example, it was shown that the FBP algorithm for CT could be translated into a neural network by mapping each mathematical operation to a network layer [47]. For parallel beam geometry, the FBP algorithm can be written as:

6 $$\begin{aligned} \hat{\varvec{x}} = \varvec{A}^T \varvec{Cy} \end{aligned}$$

with $\varvec{A}^T$ the back-projection operator and $\varvec{C}$ the convolution of the projection data with a ramp filter. When transforming this into a neural network with input $\varvec{y}$ and output $\hat{\varvec{x}}$, the first layer implements the operator $\varvec{C}$, which is readily achieved by a convolutional layer with a single one-dimensional filter of size equal to the projection size. The following layer implements the operator $\varvec{A}^T$ as a fully connected layer, but its weights are kept fixed due to memory constraints. Lastly, a ReLU activation function imposes the non-negativity constraint on the image data. This approach can be extended to fan beam and cone beam geometries by implementing additional element-wise weighting layers before the convolutional layer and by translating the back-projection operator $\varvec{A}^T$ to the appropriate geometry. The network weights are initialized to the values known from the analytical approach, so that prior to any training, a forward pass through the network is identical to the FBP algorithm. By training on known data pairs $\varvec{x}$ and $\varvec{y}$, the weights are then updated to include processes previously not accounted for in FBP. Similar to transfer learning, only a small amount of training data can already provide reconstruction improvements due to the solid starting point offered by the weight initialization. Moreover, such a network offers easy interpretation given the one-to-one mapping between analytical operations and network layers, and is less likely to give incorrect results for edge cases due to the constraints imposed by the network architecture and fixed back-projection weights. The primary downside is that the network architecture and its number of learnable parameters may be too limiting to correctly model all imperfections and noise characteristics.

Similar to how it is done for FBP, iterative approaches can also be translated into a neural network through a process commonly referred to as algorithm unrolling or unfolding. This methodology was first proposed to improve the computational efficiency of sparse coding algorithms [48], but can be extended to the iterative methods used in medical imaging. The core idea of algorithm unrolling is to fix the number of iterations, map each update $\varvec{x}^{n} \rightarrow \varvec{x}^{n+1}$ to a block of network layers $F^n$ and stack these together to form an end-to-end mapping $\varvec{y} \rightarrow \hat{\varvec{x}}$. Network parameters can then be optimized using data pairs $\varvec{y}$ and $\varvec{x}$. The mathematical formulation and therefore network architecture of the iteration blocks $F^n$ depend on the imaging modality and iterative framework, but will contain terms relating to the data consistency *f* and the regularization *R*. Parameters we are fairly confident in can be kept fixed (those relating to the data consistency) while others we are less knowledgeable about should be learned (the regularization parameters). In contrast with the original iterative algorithm, each block $F^n$ and its corresponding step size can be different and optimized with their own unique weights.

Let us discuss a specific example in more detail to obtain more insight about the unrolling process: ADMM-CSNet [49], an unrolled version of the alternating direction method of multipliers (ADMM) algorithm for use in compressed sensing MRI. In this case, we can choose the $l_2$ norm as the objective function *f* and rewrite equation 4 as

7 $$\begin{aligned} \hat{\varvec{x}} = \arg \min _{\varvec{x}} \frac{1}{2} \Vert \varvec{Ax}-\varvec{y} \Vert _2^2 + \sum _{l=1}^{L} \lambda _l R(\varvec{D}_l \varvec{x}) \end{aligned}$$

where the regularization term, consisting of *L* regularization functions, imposes an additional sparsity constraint on the reconstructed image $\varvec{x}$. That is, there must exist transformation matrices $\varvec{D}_l$, e.g., a discrete wavelet transform, so that $\varvec{D}_l\varvec{x}$ becomes sparse. The regularization function *R* can, for example, be chosen as the $l_1$ norm to promote sparsity, with the regularization parameters $\lambda _l$ determining the weight of the regularization. This equation can be solved by the ADMM algorithm by breaking the problem into smaller subpieces. Concretely, we split the data consistency and regularization updates by introducing an auxiliary variable $\varvec{z}$:

8 $$\begin{aligned} \hat{\varvec{x}} = \arg \min _{\varvec{x}} \frac{1}{2} \Vert \varvec{Ax}-\varvec{y} \Vert _2^2 + \sum _{l=1}^{L} \lambda _l R(\varvec{D}_l \varvec{z}) \quad s.t. \quad \varvec{z} = \varvec{x} \end{aligned}$$

resulting in the following subproblems to be alternately optimized:

9 $$\begin{aligned} & \arg \min _{\varvec{x}} \frac{1}{2} \Vert \varvec{Ax} - \varvec{y} \Vert _2^2 + \frac{\rho }{2} \Vert \varvec{\beta } + \varvec{x} - \varvec{z} \Vert _2^2, \\& \arg \min _{\varvec{z}} \sum _{l=1}^{L} \lambda _l R(\varvec{D}_l \varvec{z}) + \frac{\rho }{2} \Vert \varvec{\beta } + \varvec{x} - \varvec{z} \Vert _2^2, \\ & \qquad \varvec{\beta } \leftarrow \varvec{\beta } + \eta (\varvec{x}-\varvec{z}) \end{aligned}$$

with $\rho$ a penalty parameter, $\eta$ an update rate and $\beta$ a scaled Lagrangian multiplier. ADMM-CSNet unrolls these iterative updates, see Fig. 11. Each iteration block $F^n$ consists of three operations: the reconstruction layer $\varvec{X}^n$, the auxiliary variable update $\varvec{Z}^n$ and the multiplier update layer $\varvec{M}^n$ corresponding to the solution of each of the above equations. In this network, previously fixed parameters and functions are now either learnable (e.g., the penalty parameter $\rho$) or entirely replaced by a more generic operation (e.g., the transformation matrices $\varvec{D_l}$ are replaced by a convolutional layer). These can be trained in an end-to-end fashion, where the parameters are not constrained to be the same in different iteration blocks. Data consistency is still ensured by making use of the known system matrix $\varvec{A}$.

It should be noted that there is a fair amount of flexibility when it comes to how the regularization steps are implemented in the neural network, and this was only a specific example. Certain studies opt to keep the original regularization update and simply make its parameters learnable [50], whereas others replace the entire regularization update with a more generic denoising CNN [51]. The latter can be seen as a middle ground between data-driven and model-driven approaches, combining aspects of both. Several studies from CT [52, 53], MRI [50, 51, 54–59] and PET [60, 61] have shown that unrolled algorithms can improve both computation speed and reconstruction quality compared to traditional iterative methods, while offering a robust and interpretable reconstruction procedure. We refrain from going into additional implementation details during this review since the large diversity between algorithms makes it difficult to give a general yet concise overview, especially when taking multiple imaging modalities into account. Instead we refer to some other review studies more dedicated to the subject [62–64].

### Image restoration

One of the primary image degrading factors in medical imaging is noise arising from physical process randomness and scanner limitations, with possible artifacts produced by non-uniformity or incompleteness in the measurement data further reducing image quality. While the deep learning reconstruction methods discussed in section "Image reconstruction" learn to correct for these effects through training data, no such corrections are included in analytical approaches. Even iterative algorithms that include noise suppression via the regularization term may still exhibit artifacts or result in poor images when presented with limited measurement data. In these cases, deep learning can be used as a post-processing tool for restoring noisy or corrupted images. Common examples would be low-dose and limited angle tomography scans or undersampled MRI scans from which the matching high-dose and full angle acquisition or fully sampled scan is to be restored.

#### Supervised methods

Supervised image restoration requires known training pairs of low-quality images $\hat{\varvec{x}}_{L}$ (containing artifacts or high noise levels) and high-quality images $\hat{\varvec{x}}_{H}$ (artifact-free or low noise levels). A neural network *F* is then trained to map the low-quality image to its corresponding high-quality version.

10 $$\begin{aligned} \hat{\varvec{x}}_{H} = F(\hat{\varvec{x}}_{L}) \end{aligned}$$

The procedure shares many similarities with the data-driven reconstruction methods in section "Data-driven approaches," but rather than the measurement data, the already reconstructed images are used as the input. This facilitates training as the network no longer has to learn the entire imaging process, and leads to reduced data needs for good network performance. Simulations or experiments can provide the training targets $\hat{\varvec{x}}_{H}$, from which the corresponding inputs $\hat{\varvec{x}}_{L}$ are easily obtained by removing a subset of measurement data or by introducing artificial noise prior to reconstruction. A variety of network architectures can be used for *F*, of which a few examples will be discussed.

One of the simplest architectures conceivable for this task are the 3-layer deep CNNs used for limited angle CT artifact removal [65] or for low-dose CT denoising [66]. The limited angle CT network uses a full image obtained by FBP as input and removes the directional artifacts arising from the removed angles. The low-dose CT denoising network instead opts to work on patches of the image. One advantage of using patches is that many can be extracted from a single image, leading to a much larger training dataset. Additionally, if the patches are small enough, 3D convolutional networks become viable due to the reduced memory requirements, although this particular network opted for 2D convolutions. A disadvantage of using patches is the loss of long-range spatial information, which could play an important role depending on the specific noise generation procedure. Streak artifacts produced by limited angle tomography propagate throughout the whole image, whereas the noise present in low-dose scans remains more local. In both networks, all three layers are implemented as a convolution, with the first two using a ReLU activation for nonlinearity. Each layer corresponds to a specific mathematical operation: the first performs feature extraction, the second applies a nonlinear mapping suppressing those features corresponding to artifacts or noise, and the final layer recombines them into a new image. These networks have the advantage of being interpretable, but may be too constraining compared to more general, deeper networks.

The encoder–decoder design used for direct image reconstruction can again be used for image restoration, although in this case skip connections are usually added between the layers, resulting in the well-known U-Net [23] architecture seen in Fig. 8. The skip connections are essentially a copy–paste–concatenate operation where the output of early layers in the network is added to the later layers. They allow high-level features to be reused later on by bypassing other layers, thereby improving training convergence and performance. While the U-Net architecture was originally used and continues to be used for image segmentation tasks, it is nowadays also one of the more prominent network architectures in image restoration. An additional modification that is often added to the U-Net for image restoration is a residual connection between input and output. Given the structural similarity between $\hat{\varvec{x}}_{L}$ and $\hat{\varvec{x}}_{H}$, the network essentially needs to learn the identity mapping as a part of the image restoration procedure. Therefore, a residual connection is often employed (which simply adds the input to the output) so that the network only has to learn the residual noise $\hat{\varvec{x}}_{noise} = \hat{\varvec{x}}_{L} - \hat{\varvec{x}}_{H}$ rather than directly generating $\hat{\varvec{x}}_{H}$. This methodology was first proposed as a general image denoising method [67] and quickly found its way to medical imaging. Now the network only needs to find the perturbations with reference to the identity transform, a generally easier task. Such a small change can lead to large improvements in convergence and training data needs. These U-Net-based networks have been used to great success in sparse view CT [68], low-dose CT [69], converting low-count to high-count PET images [70, 71], SPECT [72], MRI denoising [73] or restoring undersampled MRI scans [74, 75].

Another possible network architecture is based on ResNet [22], where rather than employing an encoder–decoder style network with symmetrical skip connections, many residual blocks, where the output of each block is summed with its input, are appended one after another. A variant of ResNet has, for example, been used for denoising PET images in [76].

The aforementioned networks can also be adapted for other types of inputs and outputs. Some studies on limited angle tomography, for example, choose to perform image restoration in sinogram space ($\hat{\varvec{y}}_{L} \rightarrow \hat{\varvec{y}}_{H}$) prior to image reconstruction [77, 78], although both options are compared for partial-ring PET in [79], showing better results using image space data. Alternatively, dual imaging modalities such as PET/MRI may use the MRI scan as an additional input to provide anatomical information, helping with the denoising of the PET scan [80]. The relative weight that should be given to both inputs is automatically derived during the training procedure, without any need for manual tuning. It is also possible to use multiple sequential image slices as input, where each slice is a different channel, in order to incorporate some spatial information along the third dimension without resorting to 3D CNNs.

#### Unsupervised methods

Most unsupervised image restoration methods are derived from the deep image prior proposed in [81], which can be used for common tasks such as denoising, superresolution and inpainting. The authors showed that a randomly initialized CNN can itself serve as a prior for image restoration by treating the low-quality images as training labels. In this framework, a convolutional network *F* is trained to produce the noisy scan data $\hat{\varvec{x}}_{L}$ from a random input vector or image $\varvec{z}$:

11 $$\begin{aligned} \hat{\varvec{x}}_{L} = F(\varvec{z}) \end{aligned}$$

As the number of training iterations increase, the output approaches the noisy image $\hat{\varvec{x}}_{L}$. It is, however, observed that the optimization procedure leads us through a path for which, prior to reaching final convergence, the network outputs a restored version of $\hat{\varvec{x}}_{L}$ so that $F(\varvec{z}) \sim \hat{\varvec{x}}_{H}$. The authors suggested that this phenomenon likely emerges due to convolutional operations imposing self-similarity on the generated images, making it easier for the networks to learn meaningful signals rather than noise. In other words, it is possible to stop training at a point where the network has more or less learned the signal but has yet to learn the noise present in $\hat{\varvec{x}}_{L}$. The U-Net like architectures are a particularly good choice for *F*, since the skip connections allow to impose this self-similarity at various feature scales. We emphasize that this method requires a separate network *F* to be trained for each distinct image. In practice, the random input $\varvec{z}$ is usually replaced with a prior image containing additional information, such as the CT or MRI image for hybrid PET/CT or PET/MRI denoising [82]. A similar approach is taken in dynamic PET imaging, where the time-aggregated scan can be used as the input for denoising separate dynamic slices [83]. The training procedure is shown in Fig. 12.

Besides its use as a post-processing tool, the deep image prior can also be incorporated into the iterative image reconstruction procedure as a replacement to traditional regularization schemes [84, 85]. During each update step, the network is trained to generate the current image estimate $\varvec{x}^n$ from a prior image $\varvec{z}$, thereby performing a denoising step between each update. This methodology is different from the unrolled algorithms discussed in section "Model-driven approaches" as it still makes use of traditional iterative optimization steps rather than providing a single network used for end-to-end reconstruction. But compared to image restoration as a post-processing step, such an integrated approach has the advantage of ensuring data consistency on the final denoised image.

Not requiring any training data naturally offers a significant benefit, as it essentially solves one of the main difficulties in constructing good machine learning models. A downside of the deep image prior is, however, its need to be separately trained for each image, making the process rather slow in comparison to supervised approaches, which can use a single pretrained network for all images. Performance is also unlikely to match that of supervised algorithms trained for a specific noise level, but the flexibility of unsupervised algorithms nonetheless makes them an attractive option.

### Image registration

Image registration refers to the process of aligning two images so that anatomical features would spatially coincide. This is required when analyzing pairs of images that were taken at different times or taken by different imaging modalities. Traditionally, it is performed either manually by physicians or automatically by iterative approaches. Manual image registration is, however, time-consuming and conventional iterative methods remain limited in certain cases. This has led to the development of deep learning-based image registration algorithms, a broad subject deserving of its own review, see, for example, [86] and [87]. We will shortly discuss some of the most common methods.

#### Deep similarity metric

Traditional iterative approaches require a similarity metric for optimization, such as the sum of squared differences (SSD), cross-correlation (CC) or mutual information (MI). These metrics work well for unimodal image registration where images have the same intensity distributions, but perform poorly for multimodal registration or in the presence of noise and artifacts. Deep similarity-based registration aims to replace the conventional metrics with a deep learned metric better capable of handling these discrepancies between intensity distributions. It is accomplished by training a CNN classifier or regressor to predict a measure for how well the two images are aligned. The network output is then used as a similarity metric for optimization within traditional iterative approaches.

In [88], a 3D convolutional network uses cubic patches of T1- and T2-weighted MRI scans to predict a scalar score, estimating the dissimilarity between both patches. A dataset of aligned image pairs is available, from which non-aligned training examples are easily generated through random transformations. While the network is trained as a classifier, with training pairs belonging to either the aligned (label=-1) or the non-aligned class (label=1), the scalar output value between -1 and 1 is used as the deep similarity metric. Similarly, in [89] a binary classifier is trained to learn the alignment between CT and MRI patches, again using the continuous output value as the similarity score. In contrast to these classifier methods, in [90] a regressor is trained to estimate the target registration error between MRI and transrectal ultrasound images.

One common issue with these deep learned similarity metrics is that the similarity score with respect to transformation may not be sufficiently smooth and/or convex, hindering the convergence of traditional iterative approaches. This may be solved by improving the metric itself, with the study in [88] observing more convex similarity scores by training their classifier using the hinge loss rather than the cross-entropy loss. Alternatively, the optimization strategies themselves can be improved, as done in [90] where the authors propose the use of a differential evolution initialized Newton-based method for more robust optimization.

#### Reinforcement learning

As previously mentioned, reinforcement learning is an area of machine learning in which an artificial agent is trained to take subsequent actions in an environment so as to optimize the cumulative gains of some user-defined reward. For image registration, a CNN represents the agent, taking the pair of images (the environment state) as input and predicting the action that should be taken next in order to bring them closer to alignment. Possible actions for rigid transformations would be small discrete translations or rotations along specific axes. After an action, the images are accordingly updated and the next action can be predicted based on the new environment state, repeating the process until alignment is achieved. The network is trained by allowing the agent to semi-randomly explore the action space, rewarding it for actions that lead to alignment by optimizing a reward function. Most works [91–93] focus on rigid transformation since it can be represented by a low-dimensional action space, although methods [94] have been developed to translate the high-dimensional action space of non-rigid transformations to a lower-dimensional one for use in reinforcement learning.

#### Direct supervised transformation

Direct transformation methods aim to align two images using just a single transformation predicted by a neural network. Training data consist not of the aligned images themselves, but rather the transformation used to align them in the first place. In the case of rigid transformations, the network output consists of a limited set of parameters, e.g., 6 variables corresponding to translation and rotation in 3 dimensions. Some examples include [95] for the co-registration of X-ray attenuation maps with X-ray images and [96, 97] for the registration of T1- and T2-weighted brain MRI. All of these methods used synthesized ground truth labels; that is, training data were generated by applying random transformations to already aligned images. In the case of non-rigid transformations, a deformation vector field must be predicted. This makes the generation of realistic transformations more difficult, which is why many studies opt to use real alignments performed with, e.g., traditional approaches as training data. Examples include [98, 99] for brain MRI registration and [100] for cardiac MRI registration. These direct approaches are considerably faster than the aforementioned iterative methods, but remain complicated due to the lack of quality ground truth data and the high-dimensional output space of non-rigid transformations.

#### Direct unsupervised transformation

Unsupervised approaches for direct registration aim to bypass the problem of obtaining ground truth transformations by using a similarity-based loss function instead. While such a similarity metric is easily calculated, the difficulty lies in back-propagating the gradients during the training procedure. This became possible with the development of the spatial transformer network [101], a differentiable module allowing for spatial manipulation of data that can easily be inserted into existing network architectures. As such, the spatial transformer network can use the transformation predicted by the network to warp the moving image, which is then compared to the fixed image to calculate the similarity loss. Several studies [102–104] show promising results, but mostly remain limited to unimodal image registration given the difficulty in handcrafting good similarity metrics for the multimodal case.

Another option for unsupervised transformation is to use a deep-learned feature-based loss function. In [105], a convolutional auto-encoder is trained to generate a feature vector from input images. This is simply an encoder–decoder network that is trained to reconstruct the input as output, resulting in the encoder portion of the network transforming the input to a latent feature space. A moving image can then be deformed via a spatial transformer network, after which both the target and deformed moving image are passed through the encoder. The error between the two latent feature spaces then acts as the loss function and can be back-propagated to adjust the deformation performed by the spatial transformer network.

### Image translation

In certain instances, it may be beneficial or required to transform scans from one imaging modality to another. Most common is the generation of pseudo-CT images from MRI, finding its use in a few applications. The first is in MRI-guided radiation therapy [106], offering superior soft tissue contrast compared to CT-guided therapy without additional ionizing radiation. CT equivalent images are, however, still required for digitally reconstructed radiography (DRR)-based patient positioning and dose calculations and therefore need to be derived from the MRI image. A second application is for attenuation and scatter correction in hybrid PET/MRI or SPECT/MRI systems [107]. These corrections require an accurate map of the attenuation and scatter coefficients, which depend on electron density and are normally estimated from the CT image in PET/CT or SPECT/CT. The MRI image, however, does not scale with electron density and should therefore first be translated into a pseudo-CT image for use as an attenuation map. Lastly, pseudo-CT images generated from MRI may be used simply as a replacement to diagnostic CT, reducing the risks of ionizing radiation.

Although more conventional techniques such as segmentation-based or atlas-based approaches exist, each with their own merits and limitations [108, 109], deep learning approaches have been emerging as an alternative for fast and accurate pseudo-CT generation. Encoder–decoders are again the choice of network architecture for such image-to-image translation tasks, with the possibility of including skip connections given the structural similarities between CT and MR images. In [110], a modified U-Net architecture transforms MRI slices into CT slices, using MRI and CT image pairs of 18 brain tumor patients as training and testing data. The method produced an average mean absolute error (MAE) of 85 Hounsfield units (HU) compared to the original CT images, outperforming the average MAE of 95 HU from an atlas-based approach involving deformable atlas registration and patch-based atlas fusion. A similar approach was later developed for use in PET attenuation correction [111]. Pseudo-CT images were generated from MRI with a deep convolutional encoder–decoder network to identify air, bone and soft tissue, using a three-class tissue mask rather than continuous Hounsfield units as targets. The reference masks were obtained from co-registered CT scans by means of pixel intensity-based thresholding. The generated pseudo-CT image was then used for attenuation correction, providing good PET reconstructions, with average errors (compared to the CT-based attenuation corrected PET image) of less than 1% in most brain regions, outperforming two other common approaches, namely Dixon-based segmentation and anatomic CT-based template registration. A recent study [112] also evaluated pseudo-CT for the detection of structural lesions relating to sacroiliitis, observing better diagnostic performance compared to the original T1-weighted MRI scans.

While the structural information from MRI scans can be used to generate pseudo-CT images for attenuation correction in PET/MRI or SPECT/MRI, no such data are available in standalone PET or SPECT. A separate transmission scan can still be used to generate the attenuation map, although these are often undesirable due to increased scan times and radiation dose. Recent works [113–115] have demonstrated the ability of residual encoder–decoder networks to generate attenuation and scatter corrected PET images directly from the non-corrected images, foregoing the need of attenuation maps.

Image registration may also benefit from inter-modality image translations. As mentioned, multimodal registration is often complicated due to the difficulty in defining good similarity metrics, a problem which could be overcome by converting the images to the same modality as a preprocessing step prior to registration.

Oftentimes, researchers are dealing with large amounts of unpaired training data. While separate datasets of MRI or CT scans are readily available, paired datasets are much scarcer, requiring the same patient to have undergone both scans. The images must be co-registered as well, which by itself is a complicated and/or time-intensive task. To nonetheless make use of this unpaired data for training, cycle-consistent adversarial networks or CycleGANs [28] have been proposed. A CycleGAN is a specific type of GAN that aims to perform image translation when dealing with unpaired data, as is done in [116] for MRI-based PET attenuation correction. The network consists of two generators, $G_{CT}$ for the generation of CT images from MR and $G_{MR}$ for the inverse, and two discriminators, $D_{CT}$ and $D_{MR}$ which discriminate between real and fake CT and MR images, respectively, see Fig. 13. The set of MR images is passed through $G_{CT}$ to generate pseudo-CT images $\widehat{CT}$, for which $D_{CT}$ calculates a discriminative or adversarial loss. It is then passed through $G_{MR}$ to reconstruct the original MR image from the generated CT image, on which a cycle consistency loss is defined, measuring the mean squared error (MSE) between the original image *MR* and reconstructed image $\widehat{MR}$. A similar procedure is applied to the set of CT images, from which pseudo-MR images are generated. The final loss is a combination of the discriminative and cycle consistency losses, ensuring not only that the generator can produce realistic pseudo-CT images, but also ensuring that these generated images correctly match the original one. Just like in a conventional GAN, the generators and discriminators are updated alternately. The use of a cycle consistency loss negates the need for matching data pairs, which can drastically increase the size of available training datasets. A similar approach using a CycleGAN was used in [117] to generate attenuation corrected PET images directly from non-attenuation corrected images.

## Medical image analysis

A lot of AI algorithms applied in medical imaging are to improve the efficiency and accuracy of medical image analysis and even to extract information that is not (yet) perceived by human experts. Different applications can be identified being segmentation, treatment monitoring, prognosis, computer-aided detection (CADe), computer-aided diagnosis (CADx), etc. Given that a vast number of medical image analysis applications of AI have been reported, it is infeasible to cover all literature in this work. We therefore selected several important works across different commonly found anatomical application areas. This illustrates the potential and current progress of AI in medical image analysis. For more exhaustive literature surveys, we refer the reader to [118–122].

### Approaches

There are two main approaches to medical image analysis, being the more traditional radiomics pipeline and, more recently, the end-to-end deep learning algorithms. Radiomics is mostly used in limited data settings, which was primarily the case in the early days of medical image analysis with AI. In recent years, the availability of larger medical imaging datasets has increasingly resulted in a transition toward deep learning approaches. These datasets may, however, lack in generalizibilty, since data are obtained from different scanners with different resolutions and settings, posing an obstacle for use in clinical settings. This seems to be more a problem for MRI (with a wide variety of sequences) and PET/SPECT than for CT, although standardization efforts are being made for PET via the EARL accreditation program.

#### Radiomics

Radiomics refers to the extraction and analysis of large amounts of quantitative imaging features [123]. The aim is to convert medial images into quantitative mineable data and to make current radiological practice, which is often more qualitative, quantitative and standardized. In other words, many quantitative features are extracted from the 2D or 3D medical images, which can then be analyzed by machine learning algorithms to find correlations with certain disease characteristics, such as prognosis and disease type. When the relation between image features and genomic patterns are investigated, one often refers to radiogenomics [124]. The typical radiomics workflow consist of a segmentation, feature extraction and analysis step as illustrated in Fig. 14.

To extract radiomics features, the structures of interest need to be segmented. This is often done manually by an experienced radiologist or with (semi)-automatic segmentation algorithms. From these delineated structures, many features can be extracted describing its shape, volume, texture, intensities, etc. The last step is then to analyze the extracted features. This often starts by removing redundant and irrelevant features to select a minimal subset of highly predictive features with respect to the considered task. One can use specific feature selection algorithms or find the features that result in the best performance of the subsequent machine learning model. For final prediction, usually more traditional machine learning algorithms are used like random forests and support vector machines.

There are several challenges to the radiomics approach regarding imaging, segmentation, feature extraction and efficiency [125]. First of all, there is a large variety in scanners and imaging protocols between different institutions resulting in strongly differing image characteristics such as resolution, contrast, noise, slice thickness and intensity values. These differences have a strong impact on the extracted radiomics features reducing robustness and generalizability of the trained models across different centers. Therefore, standardized imaging protocols are preferred and data from different sources should be normalized both in space and intensity.

Secondly, since features such as shape are based on the segmentation masks, accurate and reproducible delineation is of crucial importance. Manual segmentation suffers from inter-reader variability and is labor-intensive, making it unfeasible for large databases. (Semi)-automatic segmentation algorithms are therefore increasingly developed. Training and evaluation of these algorithms are often done using manual delineations making the assessment of their true accuracy difficult. For this reason, consistency and reproducibility might be more important properties for radiomics analysis. To this end, manual interference should be minimized.

Thirdly, a vast amount of features can be defined and extracted. Consequently many of the extracted features can be redundant or irrelevant for the task at hand. Too many features can result in overfitting and proper feature selection is therefore very important. At the same time, the features are hand-engineered and defining the optimal features for a certain task is not straightforward. This way, important information in the medical images might be missed.

Finally, the entire pipeline of (manual) segmentation, feature extraction and analysis can be time-intensive, which is often not desired in clinical applications.

#### Deep learning

To address the above challenges associated with radiomics, there is a transition toward the use of end-to-end deep learning approaches [126]. They directly receive the medical images as input and provide at the output the desired outcome prediction. Often the workflow is still split into a segmentation and classification part to allow the prediction algorithm to focus on the relevant regions of interest. However, no manual feature extraction is necessary as the deep learning networks automatically learn the most optimal features. In both the segmentation and classification stages, deep networks can pave the way for state-of-the-art, unbiased, fast and automatic medical image analysis.

The challenge with deep learning on the other hand is the requirement of even more data to train the complex (3D) networks. Large datasets are not always available and strongly application dependent. Moreover, deep learning often lacks interpretability. In radiomics, the features used by the model to make a certain prediction can be identified and interpreted, whereas deep learning is seen as a black box. Hence, although there is an increasing use of deep learning approaches to achieve state-of-the-art performances, radiomics is still often employed when limited data are available and insight in the decision process is necessary.

### Segmentation

As discussed in the previous section, segmentation of structures of interest is an important task in medical image analysis. It is not only an important preprocessing step to improve further classification and diagnosis, it is also relevant for therapy planning and assessing therapy response. Automatic segmentation has many advantages compared to labor-intensive manual segmentation suffering from inter-reader bias and low reproducibility, and is therefore widely investigated [127–129].

Where the early segmentation systems used region-growing, clustering and traditional machine learning approaches based on handcrafted features, deep learning approaches now dominate the state of the art in medical image segmentation. The most well-known CNN architecture for medical image segmentation is the U-Net originally proposed for segmenting neuronal structures in electron microscopy stacks and cell segmentation in light microscopy images [23]. U-Nets and its modifications are the state-of-the-art architectures in many segmentation tasks.

A 3D variant of the U-Net architecture, called V-Net, was proposed in [130] with residual blocks in the encoding and decoding paths for prostate segmentation in MRI. They used a novel cost function to train the model based on the Dice score, a measure of overlap between two sets *X* and *Y*:

12 $$\begin{aligned} \text {Dice score} = \frac{2 |X \cap Y|}{|X| + |Y|} \end{aligned}$$

This allows a more balanced evaluation of segmentation performance in case the structure of interest is much smaller compared to the entire image. Since then, Dice loss is one of the most used cost functions for segmentation tasks. They trained and evaluated their model on the PROMISE12 [131] dataset of the MICCAI Prostate MR Image Segmentation challenge organized in 2012 and reached an average Dice score of 87%.

A self-configuring deep learning method for medical image segmentation, called nnU-Net was proposed in [132]. It automatically adapts preprocessing steps, network architecture (2D, 3D or cascaded U-Net), training and post-processing depending on the task and dataset properties. nnU-Net achieves state-of-the-art results in many biomedical segmentation challenges and won first place in the Medical Segmentation Decathlon [133] organized in 2018 [134]. The aim was to evaluate the generalizability of a segmentation algorithm across many different tasks instead of designing specialized solutions for one specific task. The challenge includes segmentation of 10 structures: liver, colon, pancreas and lung tumors in CT, brain tumors and prostate in multimodal MRI, hippocampus and cardiac in mono-modal MRI and hepatic vessels and spleen in CT. Several segmentation examples from the Medical Segmentation Decathlon are included in Fig. 15.

### Detection and diagnosis

Computer-aided detection consists of localizing organs or abnormalities such as lesions. It can be seen as a preprocessing step followed by further diagnosis of the found region of interest (ROI). Note that some of the discussed studies may overlap with the subject of segmentation covered in the previous section.

#### Chest pathology

One of the most widely studied topics is lung nodule detection in low-dose CT scans, which is an important step in identifying early stage lung cancer [135]. Early detection reduces lung cancer mortality and screening programs are increasingly implemented. As interpretation of lung CT scans to find small lung nodules is tedious, error-prone and time-consuming this puts a lot of pressure on radiologists. Different algorithms for automatic lung nodule detection were compared in the LUNA16 (Lung Nodule Analysis 2016) challenge [136]. This challenge made use of the publicly available LIDC-IDRI (Lung Image Database Consortium and Image Database Resource Initiative) dataset containing 888 chest CT scans with lung nodule annotations performed by four radiologists [137, 138]. Most of the proposed methods consist of two stages: a candidate detection stage and a false positive reduction stage. The candidate detection stage typically makes use of a 2D (slice-level) or 3D U-Net architecture and often has a high sensitivity at the cost of many false positives. Therefore, the false positive reduction stage additionally classifies the found ROIs as a true nodule or not using standard classification CNN architectures. Through the combination of different solutions, a sensitivity of over 95% was achieved at less than 1 false positive per scan.

To analyze screening CT scans for lung cancer, the found nodules with nodule detection algorithms need to be classified according to malignancy [135]. Many different types of algorithms have been proposed for benign–malignant pulmonary nodule classification, including more traditional radiomics approaches as well as 2D or 3D convolutional neural networks. Diagnosis of lung cancer based on low-dose CT was the topic of the 2017 kaggle Data Science Bowl [139]. The top ten submissions all used deep learning algorithms often with a similar approach as for lung nodule detection. Figure 16 shows an illustration of a typical lung cancer screening pipeline with 3D CNNs. The winning algorithm consisted of two modules: a 3D region proposal (nodule detection) network and a second module evaluating the cancer probabilities for the five detected nodules with highest detection confidence [140]. Both modules made use of a modified U-Net architecture. A few years later, Google researchers published an end-to-end lung cancer screening algorithm using [141]. They employ a 3D inflated inception architecture [142] which builds upon the inception network for 2D image classification pretrained on natural images from the ImageNet dataset [143], but inflates the filters into 3D. Their model achieves a state-of-the-art performance on the NLST (National Lung Screening Trial) dataset [144], containing 6716 cases and using an independent clinical validation set of 1139 cases. They obtained an AUC (area under the receiver operating characteristic curve, an aggregate measure evaluating model performance across the entire range of classification thresholds) of 94%, which was on par with or even outperforming six radiologists. Other applications of AI in chest pathology include diagnosis of pulmonary embolism, tuberculosis, airway diseases, interstitial lung disease and others [145].

Recently, medical imaging such as X-ray and CT have played an important role in diagnosis and management of COVID-19. Many artificial intelligence tools have been developed and contributed to improve the safety, efficiency and accuracy of the imaging workflow to fight COVID-19 [146–152]. An AI system to detect COVID-19 pneumonia in chest X-rays was proposed in [146]. After preprocessing consisting of image normalization and lung segmentation using the U-Net, a CNN was used for patch- and image-level classification. The network was pretrained to detect tuberculosis and subsequently fine-tuned to detect pneumonia in general and COVID-19 pneumonia. Evaluation on a test dataset of 454 chest radiographs from an independent Dutch hospital shows an AUC score of 81%, which was comparable to the performance of six chest radiologists. In [153], the authors aimed to introduce a standardized reporting system for CT of COVID-19. They assess the suspicion of COVID-19 infection using the CO-RADS score, a scale from 1 (very low) to 5 (very high). An AI tool to automatically asses CO-RADS score and extent of infection was proposed in [150]. The system consisted of three successively applied deep learning algorithms performing lobe segmentation, lesion segmentation and CO-RADS scoring, respectively. Pulmonary lobe segmentation was performed using a two-stage U-Net [154]. For segmentation of ground glass opacities and consolidation in the lungs, a 3D U-Net built with the nnU-Net framework [132] was used. It was trained on 108 scans with corresponding manual delineations. By computing the percentage of affected parenchymal tissue, the severity score could be assessed. To determine the CO-RADS score, again the 3D inflated inception architecture [142] was employed.

#### Breast cancer

Another well-researched use case of AI in radiology is breast cancer screening [155, 156]. Randomized trials show reduced mortality from breast cancer after mass screening with mammography, leading to a widespread implementation of screening programs. This results in an increased workload for radiologists but also a lot of data. Mammography reading, i.e., finding masses and/or calcifications and identifying them as benign or malignant, is complex and suffers from large inter- and intra-observer variations, leading to missed lesions, but also to many false positives. False positive testing leads to additional healthcare costs and emotional stress for patients and family. To reduce the error rate, blinded double-reading by two independent readers was introduced in many European countries, increasing the workload even further.

A large publicly available dataset for computer-aided breast cancer screening is the CBIS-DDSM dataset (Curated Breast Imaging Subset of the Digital Database for Screening Mammography) on The Cancer Imaging Archive (TCIA) [157–159]. It contains mammography data from 1566 participants with corresponding ROI segmentations and verified pathology information. In 2017, the digital mammography DREAM challenge was organized, aiming to develop algorithms that can improve early breast cancer detection [160]. Similarly to lung nodule analysis, most state-of-the-art CAD systems for breast cancer screening rely on deep learning algorithms and consist of a candidate detection stage and a classification stage.

In [161], the authors compared a state-of-the-art CAD system relying on manually designed radiomics features with a convolutional neural network (see Fig. 17). Both systems were trained on a large dataset of 45000 mammograms and used the same candidate detection approach. To obtain lesion candidates, a random forest classifier was trained on pixel-based first- and second-order Gaussian kernel features. An AUC score of 91% and 93% was achieved with the radiomics approach and with the CNN, respectively. Through combination of the CNN with the manual features, the performance could be improved to an AUC of 94%. Comparison with certified radiologists showed no significant difference in performance.

The first UK company receiving a CE mark for deep learning in radiology is Kheiron Medical Technologies [162]. Their mammography screening system called Mia (mammography intelligent assessment) is allowed to be used as a second reader in breast cancer screening. The deep learning algorithm was trained on more than one million screening mammography images.

#### Cardiovascular diseases

Various imaging techniques play an important role in the diagnosis and management of cardiovascular diseases (CVDs) including echocardiography, CT, MRI and nuclear medicine [163]. Artificial intelligence techniques are applied to many cardiac diagnostic applications including myocardial infarction, cardiomyopathies, coronary artery diseases, valvular heart diseases, etc. [164, 165]. An important step in the detection and diagnosis of CVD is motion tracking and segmentation of the main chambers [166–175]. This allows quantification of cardiac morphology (e.g., ventricle volumes) and cardiac function (e.g., ejection fraction and wall thickening). Therefore, continuing progress is made for cardiac segmentation enabled by several ongoing challenges such as LVQuan (Left Ventricle Full Quantification Challenge [176]) and MnMs (Multi-Centre, Multi-Vendor & Multi-Disease Cardiac Image Segmentation Challenge [177]).

In [173], an automatic method was proposed to classify cardiac pathologies such as dilated cardiomyopathy, hypertrophic cardiomyopathy, myocardial infarction and right ventricle abnormality based on cine MRI, see Fig. 18. Given two MR images from a 2D+t cine MRI sequence, apparent flow is estimated using a U-Net type network. Through combination with segmentation, time series of the radius and thickness of myocardial segments are extracted describing cardiac motion. These features are then used to diagnose cardiac pathologies with binary logistic regression classifiers. The model was trained and evaluated on the ACDC (Automatic Cardiac Diagnosis Challenge) dataset [178] and achieved an accuracy of 94%.

The use of machine learning for per-vessel prediction of early coronary revascularization after fast myocardial perfusion SPECT imaging is studied in [179]. A total of 1980 patients were included from 9 centers in the REFINE SPECT registry. A LogitBoost classifier used 18 clinical, 9 stress test and 28 imaging features to predict early coronary revascularization. Compared to standard quantitative analysis (total perfusion deficit), an improvement is achieved with the ML classifier (AUC of 79% versus 71%). The ML algorithm also outperforms expert interpretation by nuclear cardiologists.

In [180] the potential of deep learning is investigated for prediction of obstructive coronary artery diseases from SPECT myocardial perfusion imaging. The study population comprised of 1638 patients from different institutions. Compared to standard quantitative analysis, the CNN performed better with a per vessel AUC score of 76% versus 73%.

#### Abdominal diseases

Facilitated by large public datasets like the Medical Segmentation Decathlon [134] and DeepLesion [181] data sets, accelerating progress has been made in automated segmentation, detection and diagnosis of abdominal anatomies and diseases [182, 183].

A universal lesion detector in abdominal CT was developed in [181]. The authors collected a large-scale dataset composed of CT scans from 4,427 patients containing 32,120 lesions from various anatomical sites including lung, liver, lymph nodes, kidney, bone and so on. Their proposed lesion detector based on a VGG-16 backbone [184] achieves a sensitivity of 81% with five false positives per image. AppendixNet, an 18-layer 3D ResNet for detection of appendicitis on CT examinations, has been proposed in [185]. They showed that pretraining the network on a large collection of YouTube videos called Kinetics improved the performance from an AUC of 72% to 81%. The potential of deep learning for noninvasive and automatic kidney function estimation based on ultrasound has been demonstrated in [186].

#### Neurological diseases

Application of AI to neuroimaging has seen a lot of interest [187]. Possible tasks include brain age prediction [188, 189], cortical and cerebellum parcellation [190, 191], Alzheimer’s disease classification [192, 193], schizophrenia classification [194, 195], intracranial hemorrhage detection [196, 197], aneurysm detection [198–200] and others.

Cerebral aneurysms can cause subarachnoid hemorrhages and early detection is critical for management guidance. Usually CT angiography is used for cerebral aneurysm examination associated with high sensitivity. However, because of the small size of cerebral aneurysms, some may be overlooked during the initial assessment. In [200], a deep learning system was proposed for aneurysm detection with CT angiography. The detector based on an encoder–decoder architecture with convolutional block attention modules (see Fig. 19) was developed on a large dataset of 1,068 CT angiograms and evaluated on an external test set of 400 CT angiograms. They achieved a sensitivity of 97.5% and conclude that the overall detection performance of radiologists increased with the help of the algorithm.

A deep learning model to predict Alzheimer disease using $^{18}\hbox {F}$-FDG PET of the brain was developed in [201]. An InceptionV3 architecture was trained on data from the ADNI (Alzheimer’s Disease Neuroimaging Initiative) dataset [202]. The algorithm achieved an AUC of 98% with a 100% sensitivity and 82% specificity at average of 75.8 months prior to the final diagnosis.

The recent approval by the FDA (Food and Drug Administration) of Aducanumab, a drug designed to lower the amyloid plaque burden in the brain should renew the interest of the medical community for amyloid plaque PET imaging. In this regard, DL developed for quantifying amyloid burden with increased accuracy may prove of great value. Further, as several radiotracers are available for that purpose, the approach proposed by Kang et al for translating the results obtained with [$^{11}\hbox {C}$]PIB and [$^{18}\hbox {F}$]Florbetapir into one another, appears highly attractive [203, 204].

#### Whole-body imaging

Deep learning algorithms are also applied to analyze whole-body PET/CT scans [205]. In [206], different CNNs were evaluated to detect, localize and classify $^{18}$F-FDG-avid foci in whole-body $^{18}$F-FDG PET/CT images of patients with lung cancer and lymphoma. The CNNs were trained and evaluated on a dataset of 629 patients (302 with lung cancer and 327 with lymphoma). On the test set, the CNN was able to classify $^{18}$F-FDG-positive foci as suspicious or not suspicious of cancer with an AUC of 99% for lung cancer and 98% for lymphoma. The overall localization accuracy was 96.4% for the body part, 86.9% for the specific region (i.e., organ) and 81.4% for the subregion.

A follow up study evaluated the usefulness and performance of the above CNN in research and clinical routine [207]. Automatically segmented total metabolic tumor volumes of diffuse large B cell lymphoma lesions were predictive for clinical endpoints such as disease-free survival and overall survival. Yet the Dice coefficients between manual and automatic segmentations was only 0.65 in a research cohort and 0.48 in a routine cohort.

## Conclusions

We have seen that deep learning can be used in many aspects of the imaging and radiology pipeline, often outperforming traditional methodologies in terms of speed, accuracy or both. It is a quickly adapting field that has greatly been gaining traction over the past 5 years, and will likely keep doing so for the foreseeable future, with new approaches constantly being tested and developed. As both hardware and technical expertise keep improving, we can expect these networks to solve increasingly complex tasks to enable better diagnostic performance over shorter time frames.

There remain, however, several challenges to the adoption of AI in medical imaging. Although the amount of imaging data is rising fast, the number of curated datasets is still limited. Data are scattered across clinical centers with highly varying imaging protocols, recorded modalities, patient groups, included patient information, annotations, etc. Data curation and annotation of medical images is time-consuming, requires expert knowledge and is subject to inter- and intra-observer variability. It is difficult to gather enough data for rare pathologies and the distributions between different classes are often highly unbalanced. Although initiatives hosting publicly accessible medical image datasets such as The Cancer Imaging Archive [159] exist, the availability of medical imaging data to train AI algorithms is still limited, certainly when compared with natural image datasets like ImageNet [143] containing millions of images. Additionally, as these AI tools can have a direct influence on diagnosis and treatment planning, more research is necessary toward explainable AI in order to understand and trust these algorithms. While steps have certainly been taken in this direction (see, e.g., section "Model-driven approaches"), many deep learning algorithms are still seen as a black box and it is difficult to understand how and why the algorithm makes certain predictions and under what circumstances it might fail. Combined with lack of standardization of medical imaging scanners required for good generalizibility across different centers, this leads to hesitant adoption of these algorithms in routine clinical procedures.

Nonetheless, through a combination of larger, standardized datasets, a better understanding of deep learning, by both experts and the general public, and the development of explainable AI, we believe that deep learning will become increasingly common in clinical routine during the next few decades.

## Data Availability

Not applicable.

## Abbreviations

ACDC: Automatic Cardiac Diagnosis Challenge
Adam: Adaptive moment estimation
ADMM: Alternating direction method of multipliers
ADNI: Alzheimer’s Disease Neuroimaging Initiative
AI: Artificial intelligence
AUC: Area under the receiver operating characteristic curve
AUTOMAP: Automated transform by manifold approximation
CADe: Computer-aided detection
CADx: Computer-aided diagnosis
CBIS-DDSM: Curated Breast Imaging Subset of the Digital Database for Screening Mammography
CC: Cross-correlation
CNN: Convolutional neural network
CT: Computed tomography (CT)
CTR: Coincidence time resolution
CVD: Cardiovascular disease
DL: Deep learning
DOI: Depth-of-interaction
DRR: Digitally reconstructed radiography
FBP: Filtered back-projection
FDA: Food and Drug Administration
FWHM: Full width at half maximum
GAN: Generative adversarial network
HU: Hounsfield units
iFFT: Inverse fast Fourier transform
LIDC-IDRI: Lung Image Database Consortium and Image Database Resource Initiative
LOR: Line-of-response
LUNA16: Lung Nodule Analysis 2016
LVQuan: Left Ventricle Full Quantification Challenge
M&Ms: Multi-Centre, Multi-Vendor & Multi-Disease Cardiac Image Segmentation Challenge
MAE: Mean absolute error
MI: Mutual information
Mia: Mammography intelligent assessment
MRI: Magnetic resonance imaging
MSE: Mean squared error
NLST: National Lung Screening Trial
PET: Positron emission tomography
PMT: Photomultiplier tube
ReLU: Rectified linear unit
ROI: Region of interest
SiPM: Silicon photomultiplier
SPECT: Single-photon emission computed tomography (SPECT)
SSD: Sum of squared differences
TCIA: The Cancer Imaging Archive
TOF: Time-of-flight
XCAT: Extended cardiac-torso
